# Optimising Irish Fen Habitat Mapping: An Evaluation of Temporal Windowing and Spectral Features Using Google Earth Engine and Random Forest

**DOI:** 10.3390/s26206580

**Published:** 2026-10-16

**Authors:** Ciara Farrell, Yuanyuan Pu, Shane Regan, Lizy Abraham, Owen Naughton

**Affiliations:** 1 Department of Built Environment, South East Technological University, Kilkenny Road, R93 V960 Carlow, Ireland; owen.naughton@setu.ie; 2 Department of Land Sciences, South East Technological University, Cork Road, X91 K0EK Waterford, Ireland; yuanyuan.pu@setu.ie; 3 National Parks and Wildlife Service (NPWS), Department of Housing, Local Government and Heritage, 90 King Street North, Smithfield, D07 N7CV Dublin, Ireland; shane.regan@npws.gov.ie; 4 Walton Institute, South East Technological University (SETU) West Campus, Carriganore, X91 P20H Waterford, Ireland; lizy.abraham@setu.ie

**Keywords:** wetland, Google Earth Engine (GEE), habitat classification, Random Forest, Sentinel-2, temporal windowing, remote sensing

## Abstract

**Highlights:**

**What are the main findings?**

- Effective Temporal Windowing: Results suggest a targeted growing-season window (June–September) captures critical phenological variation across habitat types more effectively than full-year annual composites or individual single-date scenes.
- Data-Efficiency Under Sampling Constraints: A GEE-based Random Forest classifier maintained high within-site accuracy even when training data were reduced to a 50:50 split.

**What are the implications of the main findings?**

- Targeted Field Sampling Efficiency: The stability of the 50:50 split indicates that field sampling efforts can be reduced without compromising within-site mapping accuracy, provided habitat classes are adequately represented.
- Reproducible Spatial Framework: Sampling Sentinel-2 imagery on a 5 m evaluation grid in GEE offers a reproducible, cost-effective workflow for mapping fragmented wetlands while preserving native 10 m reflectance values.

**Abstract:**

High spectral similarity and overlapping habitat boundaries present a substantial challenge for mapping and monitoring Ireland’s ecologically vital fen ecosystems. This study establishes a reproducible remote sensing workflow by combining Google Earth Engine (GEE) with Sentinel-2 imagery for Irish fen classification. This study uses a Random Forest machine learning model to evaluate five temporal windows against three spectral feature sets across three distinct sites. The results indicate that a growing season window (June–September) incorporating both bands and spectral indices captures peak phenological information more effectively than annual composites, producing high within-site, pixel-level overall accuracy across all study sites (Scragh Bog: 95.24%, Liskeenan Fen: 94.13%, Ballymore Fen: 94.43%). Although raw spectral bands achieved marginally higher localised accuracies at Liskeenan (+0.24%) and Ballymore (+1.65%), the combined feature set was maintained as the standard workflow to ensure cross-site stability, biophysical parameterisation, and resilience against localised soil background noise. To accommodate narrow, fragmented geometries, training data was extracted via pixel-level stratified sampling; while this introduces spatial autocorrelation, classification outputs showed coherent within-site spatial patterns, although accuracy estimates remain subject to spatial autocorrelation. Crucially, the Google Earth Engine-based Random Forest model maintained stable within-site classification performance when reducing the training-testing ratio from a standard 80:20 split down to a highly constrained 50:50 split. Ultimately, this workflow provides a cloud-based framework for monitoring vulnerable wetland ecosystems under real-world geometric and ground-truth data limitations.

## 1. Introduction

Wetlands are a defining feature of the Irish landscape. These ecosystems play a vital role in biodiversity, water purification, carbon sequestration, and flood regulation [1,2,3,4]. Among wetland ecosystems, fens represent a highly valuable yet vulnerable natural habitat. Fens are a type of peat-forming wetland, fed mainly by groundwater, that maintain a water table at or near the surface year-round [5,6]. Fen classification relies primarily on hydrological input, which determines both habitat structure and ecological characteristics [6,7].

Despite their ecological importance, Irish wetlands, including fens, are experiencing severe degradation and pressure from human activities such as agricultural drainage, unsustainable grazing, eutrophication, and climate change [8,9,10]. Wetlands are characterised by high structural complexity and fragmentation [11]. This is particularly evident in fens, where ecological gradients result in continuous ecotones and transitional zones between vegetation communities rather than distinct boundaries. Consequently, accurately mapping and monitoring these ecosystems poses significant challenges for environmental management and remote sensing (RS).

Establishing an accurate and repeatable protocol provides a foundation for effective ecosystem monitoring [12]. It also facilitates proactive conservation methodologies required under domestic and international regulatory requirements [13,14,15]. Traditional habitat mapping has mostly relied on manual field surveys conducted by ecologists [16]. Although highly precise, this approach is resource-intensive, faces logistical restrictions and is susceptible to adverse weather [17,18]. Ireland currently faces a shortage of specialist ecologists [19]. As a result, traditional fieldwork cannot keep pace with the rapid environmental monitoring required to track environmental change and meet regulatory reporting requirements.

Technological advancements have allowed RS to reduce on-site limitations [20,21,22]. Satellite imagery has become a cornerstone of ecosystem monitoring, providing an essential source of temporal and spatial data [23,24,25]. This transition introduces spectral challenges, such as spectral overlap [26,27]. Fen habitats may contain similar species, and their spectral signatures may closely overlap, making them difficult to distinguish. Relying solely on raw spectral bands therefore often fails to differentiate these distinct habitats, resulting in land cover mapping misclassifications [28]. Incorporating spectral indices alongside raw band data provides additional information needed to classify habitats more accurately and reduce mapping errors [13,29].

Large-scale satellite habitat mapping is primarily constrained by data volume and the need for high-performance computing infrastructure [30,31]. Google Earth Engine (GEE) offers a solution to this issue by providing an accessible cloud-based computing platform to researchers [32]. Readily available satellite imagery can be analysed using the GEE platform without the need to download imagery to local machines [33,34,35]. Machine learning (ML) algorithms provide a standard analytical framework for decoding complex, high-dimensional ecological datasets [36,37,38]. These algorithms efficiently process and decipher complex, non-linear relationships without relying on parametric assumptions. This approach can achieve high classification accuracy and protect the models against outliers [39]. Random Forest remains a predominant algorithm in remote sensing and ecology, balancing high classification accuracy with low computational overhead and strong performance on limited field training data [40,41,42]. Developed by Breiman, RF is a powerful ML algorithm that combines multiple decision trees to make predictions [43]. RF is widely used because of its robust generalisation capabilities and efficiency in handling high-dimensional geospatial datasets [44,45].

While remote sensing tools such as Sentinel-2, cloud-based platforms, and ML classifiers are well-established, a significant research gap remains in defining optimal data composition strategies for complex fen habitats. Standard remote sensing methodologies increasingly rely on annual or multi-year temporal composites to mitigate cloud cover and capture seasonal phenology [46,47,48]. However, this broad temporal averaging frequently suppresses critical seasonal variations. While specific phenological windows can highlight distinct spectral signatures, how temporal selection and training-data limits interact in fragmented landscapes remains understudied. We address this gap by evaluating temporal windows and data constraints across complex Irish fen habitats.

This study investigates the viability of a satellite-based RS workflow for fen ecosystem mapping. Specifically, this paper addresses four core objectives: (1) Assess the impact of temporal windowing on habitat classification, (2) Evaluate the predictive power of input feature configurations, (3) Determine model sensitivity to data constraints and split ratios, and (4) Discuss the implications and practical viability of the workflow for real-world ecological reporting. The aim is to establish a reproducible and consistent methodology for accurately mapping fen ecosystems using the findings from this workflow.

## 2. Materials and Methods

### 2.1. Study Sites

**Figure 1:**
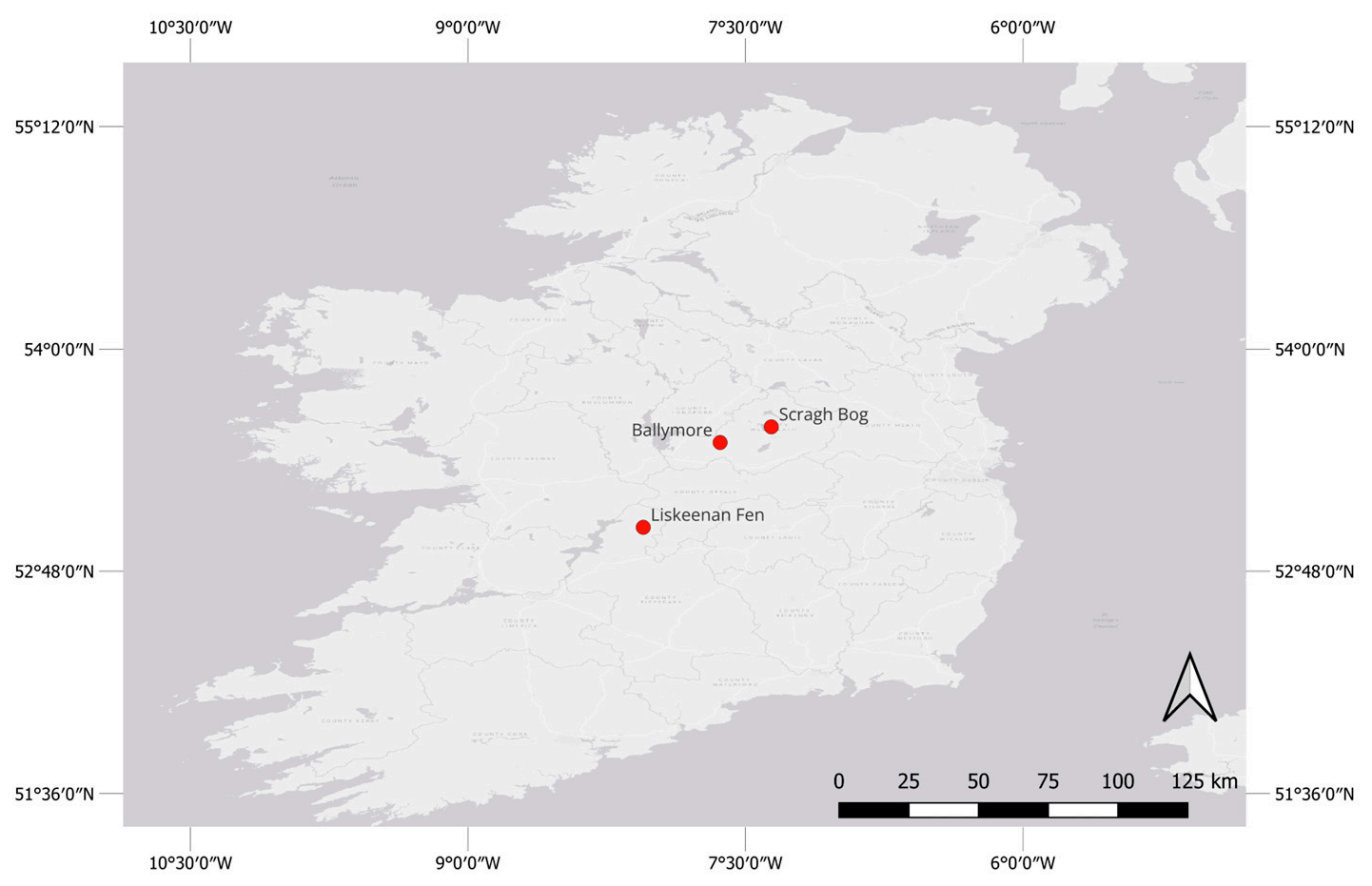
Geographic distribution of the selected study fens across the Irish midlands and mid-west regions.

This study evaluates three ecologically significant fen wetland ecosystems within the Irish midlands and mid-west: Scragh Bog (Scragh), Liskeenan Fen (Liskeenan) and Ballymore Fen (Ballymore) (Figure 1). The distribution and ecological significance of these sites are intrinsically linked to Ireland’s temperate climate, characterised by year-round precipitation and high humidity. This precipitation maintains the waterlogged conditions necessary to sustain these ombrotrophic and minerotrophic ecosystems. All sites are designated as Special Areas of Conservation (SAC) under the European Union Habitats Directive (92/43/EEC), and legally protected due to the presence of rare, vulnerable, or representative wetland habitat types listed under Annex I of the EU Directive [49].

Note that the localised site areas reported in this study represent the spatial extent of the compiled reference vegetation polygons used for modelling and testing, rather than the complete legal borders of the statutory Special Areas of Conservation (SACs). For context, the legal boundaries designated by the National Parks and Wildlife Service (NPWS) cover 42.71 ha for Ballymore Fen SAC, 43.68 ha for Liskeenan Fen SAC, and 23.85 ha for Scragh Bog SAC. Ground truthing and field sampling were strictly restricted to the core SAC perimeters, while outer non-SAC reference labels relied on remote sensing and desktop classification.

Scragh, Co. Westmeath (26.7 ha; 53.579° N,7.3600° W): This site is regarded as a prime example of an Irish wetland ecosystem which has progressively transformed from an alkaline fen to a raised bog. This rare transition supports a diverse array of vegetation and invertebrate species.

Liskeenan, Co. Tipperary (43.5 ha; 53.0363° N,8.0527° W): A calcareous fen with a hydrological system similarly found in turloughs. Seasonal inundation occurs primarily in winter, driven by groundwater dynamics associated with local swallow holes. Its mosaic of habitats, including dry grasslands and transition mires, acts as a vital refuge for rare flora.

Ballymore, Co. Westmeath (21.8 ha; 53.4946° N,7.6372° W): Chosen for its protection of transition mires and quaking bogs and a combination of habitats, this site acts as a sensitive transitional wetland ecosystem. This site is highly sensitive to hydrological shifts and plays a vital part in maintaining regional biodiversity by connecting rich fens and ombrotrophic bogs.

Baseline vegetation data, spatial distributions, and structural habitat information were derived for each site from national ecological surveys and technical frameworks established by the National Parks and Wildlife Service (NPWS), drawing on methodologies and spatial datasets outlined in the Scoping Study and Pilot Survey of Fens [50]. Habitat categories across the study areas were mapped following standard Irish heritage guidelines, using the Fossitt Level 3 classification scheme [51]. A comprehensive presence/absence matrix of all identified Fossitt Level 3 habitat categories and transitional mosaics was created and is provided in Appendix A. Table A1. Across all three study sites, a total of 23 distinct habitat polygons were delineated. The numbers of polygons per class are detailed in Table A1.

Field mapping used a minimum mapping unit of 400 square metres for each polygon based on high-resolution aerial imagery with a 2 × 2 m relevé used for delineating transitional zones on site. Field surveys were conducted along structured zigzag transects using hand-held devices and vegetation identification maps. To account for natural habitat mosaics and transitional boundaries, surveyors recorded percentage cover scores for every habitat type within a polygon to the nearest 5%, ensuring all recorded classes summed to 100%.

High-resolution orthomosaics were generated from drone surveys conducted across the study sites (with an average camera location error of 1.74 cm). Polygons were manually delineated by expert surveyors identifying homogenous vegetation patches based on dominant species composition and hydrological indicators following the Fossitt habitat classification scheme.

Field observations were carried out for Scragh on 6 September 2019 and Liskeenan on 15–16 July 2020. Exact dates for Ballymore during this period were unavailable in field records. Corresponding Sentinel-2 image acquisitions were filtered for the years 2019–2020 to match these survey ranges to minimise phenological discrepancies.

### 2.2. Data Sources and Processing

Sentinel-2 surface reflectance harmonised imagery (COPERNICUS/S2_SR_HARMONIZED) data for the years 2019 and 2020 were acquired through the GEE platform. Computations were executed via the web-based JavaScript code editor utilising the core Google Earth Engine API. This workflow, filtered spatially by site boundaries (filterBounds) and temporally across distinct windows (filterDate), allowed for the retrieval of bottom-of-atmosphere (BOA) reflectance data, which was subsequently aggregated into median composites.

We chose not to apply pixel-level cloud masking (e.g., SCL or QA60). Instead, cloud and shadow mitigation relied on scene-level metadata filtering and manual collection pruning, using median temporal aggregation to reduce residual atmospheric noise.

For the baseline annual windows (Windows 1 and 2), all available scenes across the year were ingested to maximise multi-temporal observations, supported by manual inspection to eliminate major seasonal image anomalies while relying on pixel-level median aggregation to suppress transient cloud contamination. In contrast, Windows 3, 4, and 5 applied a strict scene-level cloudy_pixel_percentage filter (<30%) prior to collection stacking, alongside manual verification to exclude corrupted scenes. Scenes exhibiting residual cloud cover or shadows over the study area were excluded from further analysis. Clean, verified observations were then compiled into their corresponding windows. The Pilot Fen Survey 2019–2020 data provided by the National Parks and Wildlife Service (NPWS, Ireland) served as reference data. All analyses were conducted in the Universal Transverse Mercator (UTM Zone 29N, EPSG:32629) projection. In accordance with GEE’s architecture, default nearest neighbour interpolation was utilised to extract pixel values onto the 5 m evaluation grid, without applying any external super-resolution or algorithms.

Five distinct composite groupings were established to represent various temporal windows, seasonal ranges, and cloud cover thresholds. A summary of these configurations and the associated number of variables utilised in this study is presented in Table 1. For windows 1 through 4, a median composite was generated by sorting all available pixel values for each individual spectral band independently over time—from lowest to highest reflectance—and selecting the median value for every pixel within the chosen temporal window. This pixel-based approach effectively removes cloud, cloud-shadow, and atmospheric outliers, yielding a clean, representative surface reflectance value for each band (e.g., blue, green, red, and near-infrared) across the seasonal window. This method was preferred over mean aggregation due to its robustness against outliers, cloud contamination, and atmospheric distortions. Since window 5 consists of a single-date acquisition, the median compositing process was unnecessary.

The five distinct temporal windows contain the following dates: Window 1 (Full year 2019: 1 January to 31 December), Window 2 (Full year 2020: 1 January to 31 December), Window 3 (Low cloud cover (<30%) 2020: 1 January to 31 December), Window 4 (Growing season 2020, 1 June–30 September) and Window 5 (Best single scene per site with lowest cloud cover, selected from the search period of 1 January 2019, to 31 December 2020)).

The best single scene per site temporal window was created using the single scene with the lowest cloud cover for each site, applying a threshold of cloudy_pixel_percentage <30 alongside visual inspection to eliminate residual cloud shadows.

For the Best single scene per site with the lowest cloud cover (2019–2020) composites, the optimal scene acquisition over the Scragh site was captured on 15 April 2020, Liskeenan was 20 September 2020 and Ballymore was 18 September 2019.

Eight auxiliary variables were incorporated into the study, consisting of spectral indices derived from the Sentinel-2 bands. These indices were specifically selected to enhance habitat classification accuracy by capturing phenological and structural surface characteristics of the sites [52,53]. While these indices share underlying bands, they were chosen to systematically evaluate whether structural variations in index formulations influence tree-splitting behaviour within the classifier. Several of these metrics (NDVI, RVI, NRVI, and SAVI) are highly collinear or represent monotonic transformations of the same underlying near-infrared-to-red reflectance ratio. Furthermore, their frequent application across the evaluated literature establishes a standardised and comparable analytical framework.

A detailed summary of all variables and spectral bands employed in this study is provided in Table 2. Note: For the Soil Adjusted Vegetation Index, a soil brightness correction factor (L) of 0.5 was used.

### 2.3. Methodology

**Figure 2:**
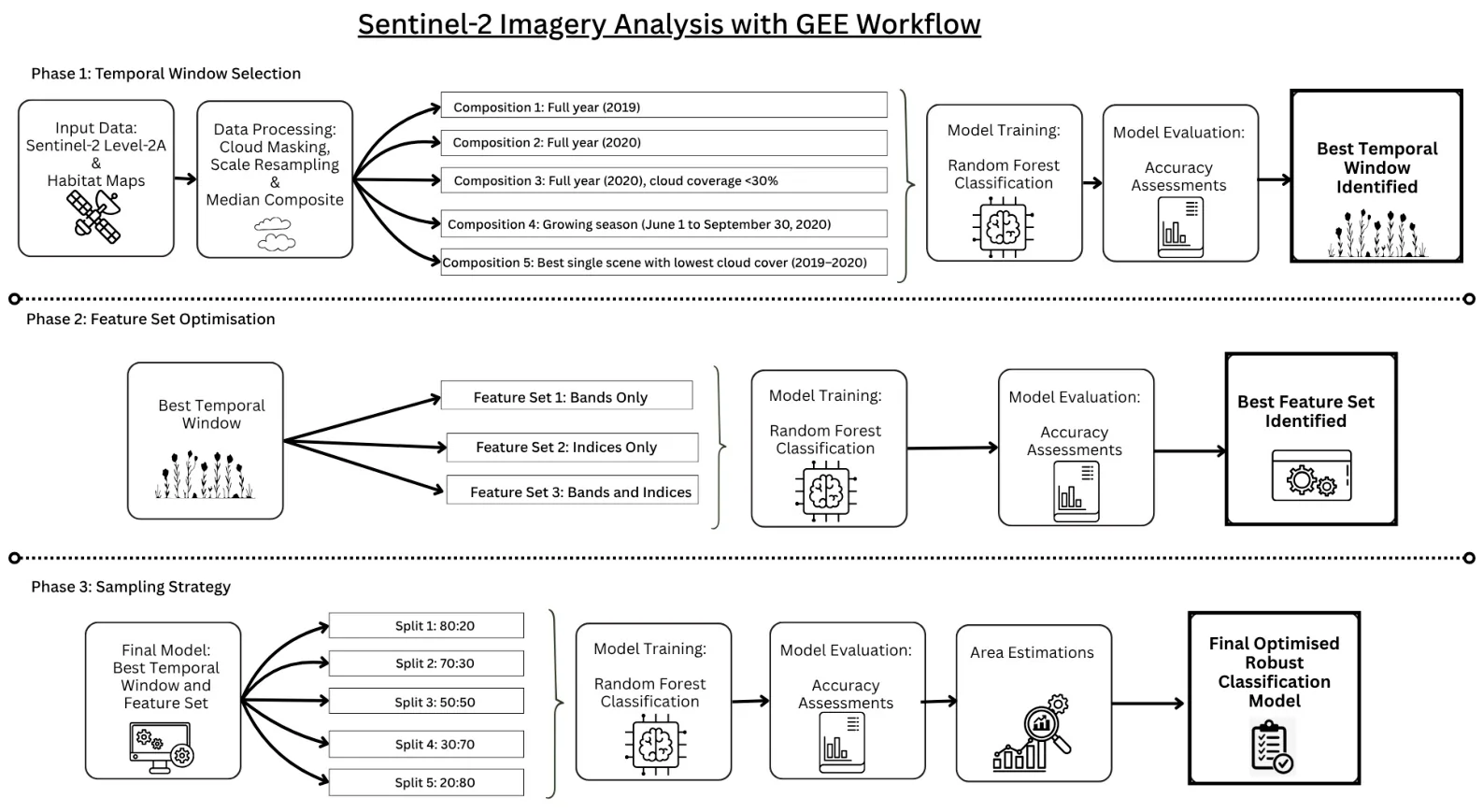
Methodological workflow for Sentinel-2 data processing and habitat classification.

The workflow followed a sequential, three-step process designed to isolate the most efficient parameters for habitat mapping (Figure 2). Specifically, phase 1 investigates the optimal temporal window for fen habitat mapping, phase 2 evaluates the combination of spectral indices within that optimum temporal window, and phase 3 assesses the impact of training data. To maximise the spectral differentiation between habitat types, the composite windows (Table 1) were selected to capture phenological variations, allowing the classifier to distinguish between similar habitat structures. Image classification and region sampling were executed within GEE. Atmospheric interference and cloud contamination were mitigated using a dual approach, depending on the temporal window. As outlined in Section 2.2, Windows 3, 4, and 5 applied a strict scene-level cloudy_pixel_percentage filter (<30%) prior to stacking, alongside manual verification.

Conversely, for Windows 1 and 2, which retained a higher density of annual scenes, cloud mitigation relied on image-level screening and manual inspection to exclude severe regional anomalies.

To improve the extraction of training data from highly fragmented and narrow polygons, the sampleRegions() function in Google Earth Engine was applied with a pixel scale of 5 m. While Sentinel-2 native spatial resolution is 10 m, specifying a finer 5 m scale enables more flexible sampling within small and irregular polygons. Operating at a 5 m sampling interval is a recognised approach in remote sensing workflows to align finer spatial grids; as no super-resolution algorithm was applied, this procedure constitutes nearest neighbour resampling rather than true physical downscaling.

The sampling strategy was deliberately implemented to increase sample size within highly restricted polygon geometries and to reduce boundary corruption from mixed edge pixels. However, we acknowledge that this pixel-level extraction, combined with random data partitioning, results in spatial dependencies between neighbouring pixels. The final pixel-level dataset was partitioned using a stratified random sampling approach to ensure representative class distributions in both the training and testing datasets. Low polygon habitat representation and structural site differences prevented true spatially independent cross-validation, such as holding out entire polygons or evaluating cross-site transferability. Consequently, the reported accuracy metrics reflect pixel-level, within-site performance influenced by spatial autocorrelation.

To determine the most effective configuration for maximising classification accuracy, three distinct feature sets were constructed featuring a combination of spectral and auxiliary variables. This approach allows a systematic evaluation of how varying feature combinations such as spectral bands, indices, or spectral bands and indices influence performance. A cross-evaluation of the five composite windows and three feature sets was performed to determine the model configuration that yielded the highest classification accuracy.

To evaluate the stability of the classification model against a reduction in input data volume, a controlled data-split sensitivity analysis was conducted. Model performance was benchmarked using five training-testing partitioning strategies: 80:20, 70:30, 50:50, 30:70, and 20:80. Furthermore, it is acknowledged that utilising the same data partitions for both hyperparameter selection and final performance estimation introduces potential selection bias.

### 2.4. Machine Learning Algorithm

The GEE platform supports several supervised classification algorithms frequently applied in ML [59]. In this study, the RF classifier was employed due to its robustness in handling complex, multi-source remote sensing data. RF is considered one of the most commonly used algorithms in research for habitat classification. The RF algorithm is widely utilised in remote sensing applications due to its robust handling of high-dimensional datasets [60] consistent performance across varying sample sizes [61].

The RF algorithm is a tree-based learning model, where each prediction is determined by aggregating the outputs of multiple independent decision trees through a majority voting system [62]. This reduces the risk of overfitting, thereby enhancing the model’s overall prediction accuracy and stability [63,64]. Two hyperparameters were configured to improve classification performance: ntree (the number of trees) and mtry (the number of predictor variables randomly selected at each node split) [65]. In this study, the smileRandomForest algorithm was configured with 250 trees, utilising Google Earth Engine’s (GEE) deterministic pseudo-random stream via randomColumn() with a fixed seed value of 42. The number of predictor variables randomly sampled at each node split was set to the default value, the square root of the total number of input features.

### 2.5. Model Accuracy Assessment

Determining the efficiency of the classification model fundamentally relies on its accuracy assessment. To evaluate classification performance, a confusion matrix was generated by comparing the classified results with the reference (ground-truth) data [66]. From this matrix, key metrics were derived to assess global and class-level performance [67,68,69]. Overall Accuracy (OA) was calculated as the proportion of correctly labelled pixels out of all predictions made [32]. Specifically, OA was calculated based on the test set as the proportion of correctly classified pixels among all test samples (Equation (1)).

(1) $$Overall\;Accuracy\;\left(OA\right)=\frac{Number\;of\;Correctly\;Preditced\;Samples}{Total\;Number\;of\;Samples}$$

Additionally, Producer’s Accuracy indicates how successfully the model identifies reference pixels for each specific class [70]. Furthermore, the kappa coefficient (κ) was utilised as a statistical measure of global agreement across the entire matrix. The κ values range from 0 to 1, where a value of 1 represents a perfect agreement and a value of 0 indicates agreement no better than random chance [71]. In addition to OA and κ, per-class performance of the final model was evaluated using Producer’s Accuracy, User’s Accuracy, and F1-score.

Following the classification, the total habitat area derived from each model configuration was compared against reference data to assess the accuracy of landscape-level quantification. These area estimates were evaluated across all five partitioning strategies, to determine performance stability and sensitivity to reduced data availability. To reduce isolated pixel noise and improve map clarity, a post-classification majority filter (3 × 3 window) was applied to the final raster maps before calculation and visualisation.

A stratified random split was implemented, maintaining a separate random data partition within each habitat class. This ensures that every class, regardless of total spatial extent, is represented in both training and testing datasets. Stratified random sampling is widely recognised in remote sensing image classification for managing class imbalance and ensuring adequate representation of minority classes [11,27,29,33,72].

The sampled data were partitioned prior to modelling: an 80% subset was allocated for training the RF classifier, while the remaining 20% per habitat class was held out as an independent, within-site test set. To ensure complete cross-model consistency, this identical 80:20 partition was fixed and utilised throughout the entire experimental sequence: first, as a baseline to evaluate and compare the five temporal windows and three feature sets, and subsequently, to benchmark the final selected under systematic reductions in training data volume. Since these test samples were drawn from the same mapped habitat polygons as the training data, performance metrics reflect within-site accuracy and remain subject to spatial autocorrelation.

### 2.6. Spatial Autocorrelation Considerations

Due to the fragmented nature of the study sites, rare habitat classes are represented by limited discrete polygons. To ensure that all habitat classes are represented simultaneously in both training and testing phases, a pixel-based stratified random sampling approach was implemented. It is acknowledged that pixel-level partitioning with contiguous polygons introduces spatial autocorrelation which inflates accuracy and κ values. These metrics represent the configured performance baseline across the three localised study sites, rather than a definitive measure of cross-regional generalisability.

## 3. Results

### 3.1. Evaluation and Validation of the Temporal Windows

To establish an analytical baseline, model performance was evaluated across all study sites to determine the optimal temporal window. A systematic evaluation was carried out across all five temporal windows and three feature sets per site. An 80% data subset was allocated for model training, while the remaining 20% was held out as an isolated, within-site test set. This 80:20 split represents the initial fixed partition described in Section 2.5. The overall classification accuracy across temporal windows and feature sets for Scragh, Liskeenan, and Ballymore are presented in Table 3, Table 4, and Table 5, respectively.

The OA results demonstrate consistent within-site classification performance across all three study sites. While specific accuracy values vary depending on the temporal window and feature set, the proposed framework yields high-pixel level accuracy, though these results are subject to spatial autocorrelation.

While OA values fell primarily in the 90% range across most configurations, exceptions were observed for the spectral indices-only models’ windows at Scragh, which resulted in lower accuracies of 64.02% for Full year 2019 and 68.78% for Full year 2020.

The results suggest that temporal window selection notably influences model performance stability, with growing-season and low-cloud composites yielding more consistent high-accuracy classifications than annual or single-scene inputs. Specifically, datasets restricted to the Growing season (2020) and Low cloud cover (<30%) (2020) windows resulted in the highly stable numerical accuracy scores. Furthermore, the Best single scene per site with lowest cloud cover (2019–2020) window, while computationally efficient, displayed a slightly lower performance, suggesting that temporal information captured over a larger time scale is most effective for classification.

To rigorously assess whether choice of temporal window significantly impacts model performance, a Wilcoxon Signed-Rank test was conducted. This test evaluated paired Producer accuracy metrics across individual habitat classes between the Low cloud cover (<30%) (2020) and the Growing season (2020) temporal windows (utilising the combined bands and indices feature set) at each study site, where the sample size (*n*) represents the number of directly matched habitat classes compared per site (Scragh *n* = 9, Liskeenan *n* = 15 and Ballymore *n* = 8).

Differences in classification performance across temporal windows were not statistically significant at Scragh (*p* = 0.910) or Liskeenan (*p* = 0.930). In contrast, Ballymore exhibited a non-significant trend approaching the α = 0.05 threshold (*p* = 0.055). This variation is likely driven by Ballymore’s complex landscape composition, where mixed habitats and subtle transitional zones increase spectral overlap between classes.

Due to spatial autocorrelation, the resulting *p*-values must be interpreted with caution regarding statistical independence. As none of these *p*-values cross the threshold of standard significance, these tests indicate that the performance differences between the two windows are numerical rather than statistically significant. Although the Wilcoxon tests did not show statistically significant differences between the low-cloud and growing-season windows at the conventional α = 0.05 threshold, the growing-season window was retained as the preferred operational configuration. This choice is justified by its high numerical performance, reduced seasonal noise, clearer phenological interpretability, smaller image volume, and lower computational demand. Therefore, the growing-season window should be interpreted as a practical and ecologically meaningful selection rather than as a statistically superior alternative.

**Figure 3:**
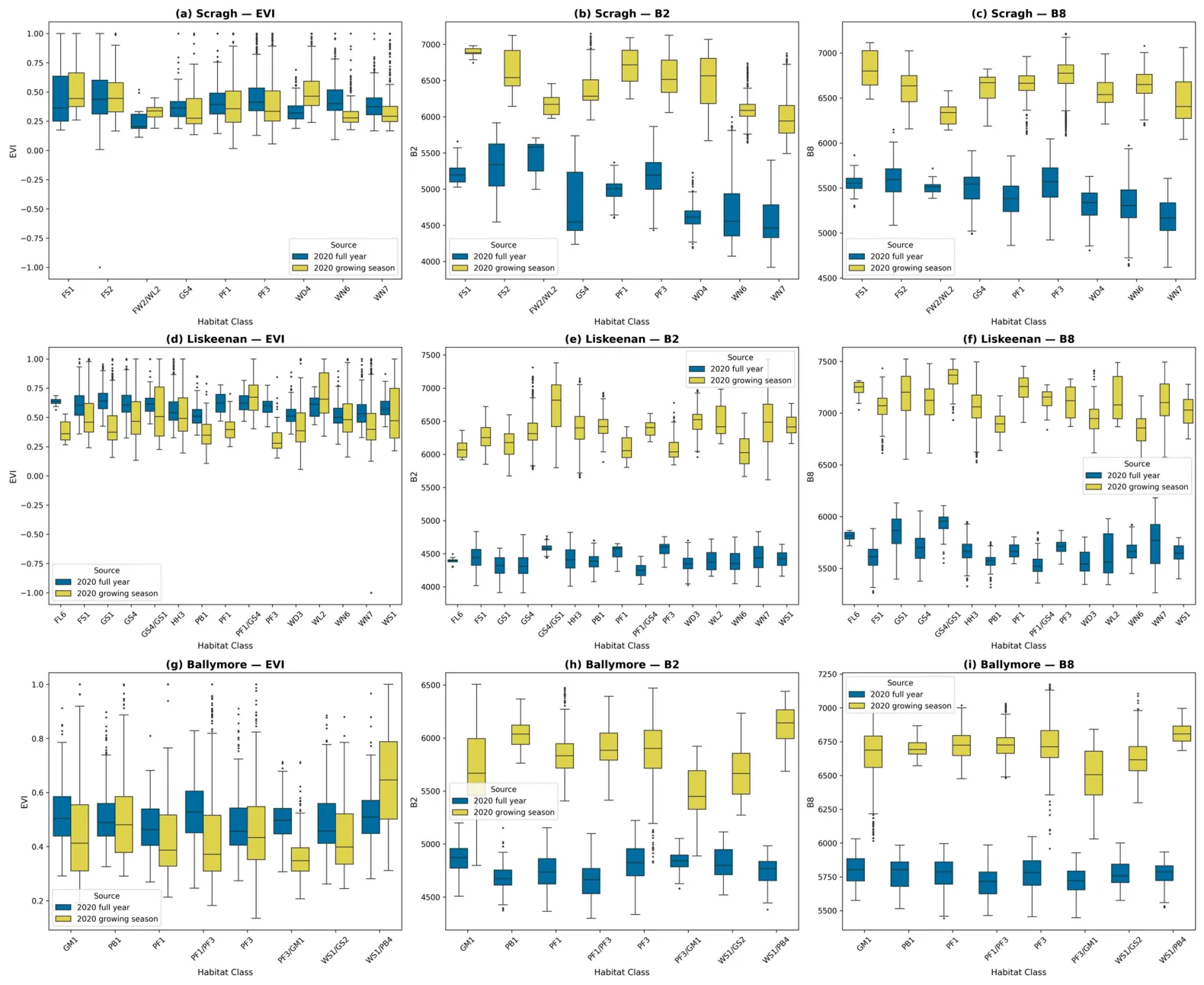
Spectral band and index comparisons between the 2020 full-year composite and the 2020 growing season composite across habitat classes for each study site: (**a**) Scragh—EVI; (**b**) Scragh—Band 2 (Blue); (**c**) Scragh—Band 8 (NIR); (**d**) Liskeenan—EVI; (**e**) Liskeenan—Band 2 (Blue); (**f**) Liskeenan—Band 8 (NIR); (**g**) Ballymore—EVI; (**h**) Ballymore—Band 2 (Blue); and (**i**) Ballymore—Band 8 (NIR).

To further investigate the similarities in classification accuracy between Low cloud cover (<30%) (2020) and Growing season (2020), spectral box plots were generated for three key features across all study sites (Figure 3)-Enhanced Vegetation Index (EVI), and bands B2 (Blue) and B8 (NIR). Note that the absolute reflectance values for the B2 (blue) band fall within an elevated range (4000–7000). This baseline inflation is attributed to Rayleigh atmospheric scattering and residual thin cloud haze resulting from the absence of pixel-level cloud masking, highlighting a key characteristic of the unmasked dataset evaluated.

As illustrated in Figure 3, the inclusion of annual data substantially reduces the spectral range of habitat classes compared to the Growing season (2020). The full-year data from the Low cloud cover (<30%) (2020) (blue) exhibits lower variance and compressed interquartile ranges. This may suggest that incorporating full-year imagery, including dormant-season data, introduces seasonal noise or spectral ambiguity, which could increase the difficulty of distinguishing between subtle habitat classes. In contrast, the summer season (yellow) reveals distinct class distributions confirming that this period captures the physiological and structural differences between habitats. By restricting analysis to the growing season, the spectral noise associated with the habitat’s dormant period is reduced.

### 3.2. Feature Set Evaluation

To address the performance of the different feature set configurations, classification accuracy was compared across all three study sites using the Growing season (2020) temporal window (Table 6). Identifying the most effective feature set is essential for increasing model accuracy across all sites. The bands and indices feature set recorded the maximum OA at Scragh (95.24%), whereas the bands-only configuration yielded the maximum values for Liskeenan (94.37%) and Ballymore (96.08%). At Liskeenan, raw bands and the combined stack achieved near-identical accuracy (a difference of 0.24%), indicating functional parity between the two configurations. Although raw spectral bands achieved the highest OA at Liskeenan and Ballymore, the combined feature set demonstrated consistently strong performance across all sites, confirming its cross-site stability. Furthermore, incorporating vegetation and water indices provides crucial biophysical parameterisation such as canopy structure and surface moisture that raw spectral bands alone cannot explicitly encode, ensuring the model remains resilient to localised soil background and atmospheric noise.

**Figure 4:**
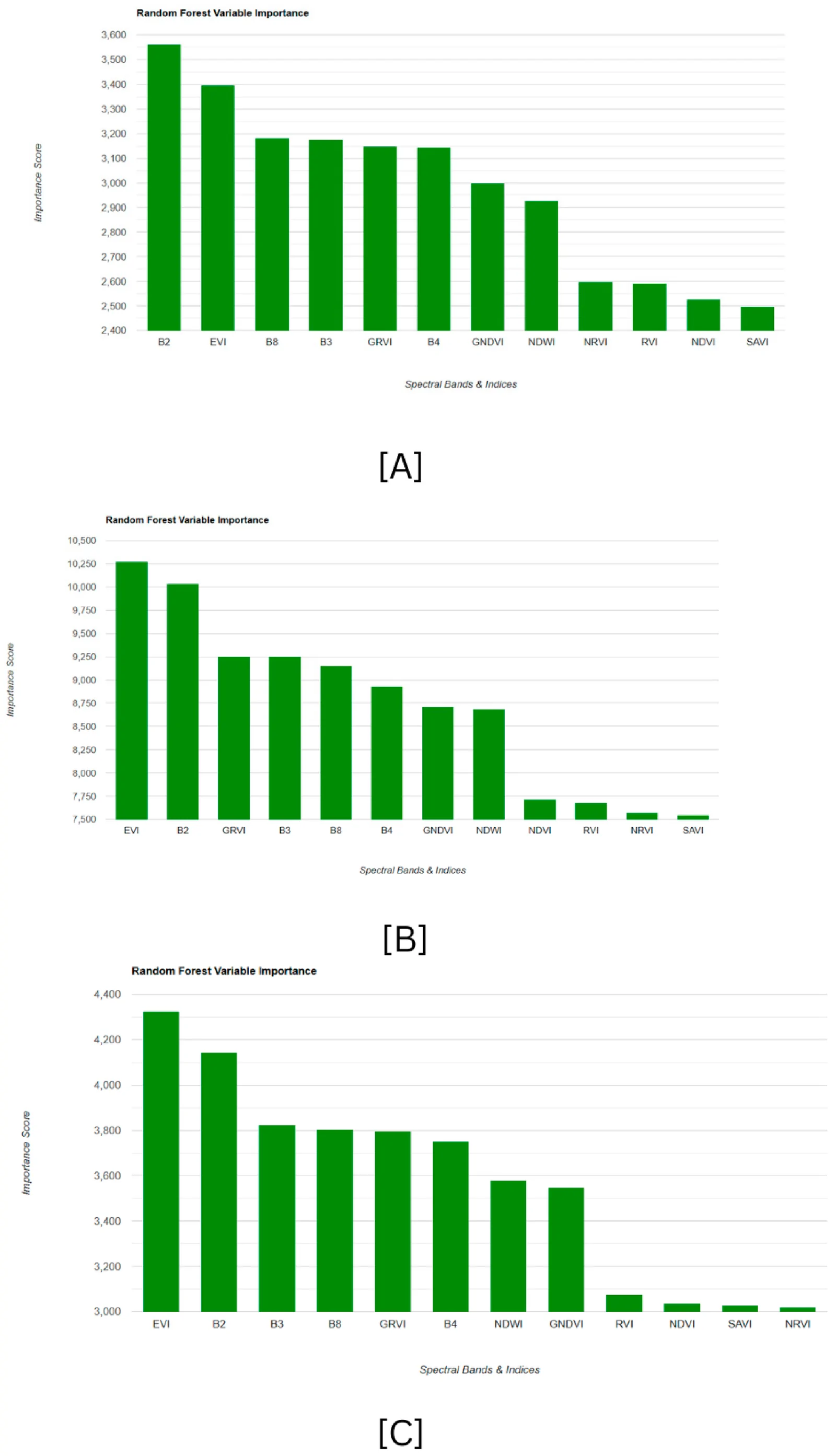
RF model feature-importance plots for the study sites: (**A**) Scragh, (**B**) Liskeenan, and (**C**) Ballymore.

To pinpoint the most important features, a RF variable importance rank was generated (Figure 4). Across the evaluated sites, the model leveraged a complementary combination of raw spectral bands and vegetation indices to discriminate fen habitats. Across all locations, B2 and EVI consistently emerged as the two highest-ranked features. Following these primary predictors, variables such as B3, B8, GRVI, and B4 displayed highly comparable importance scores, with B3 consistently placing within the top four features across all evaluated sites. Given inherent predictor correlations, these rankings reflect qualitative relative preferences rather than isolated, independent contributions. Site-level variations in feature importance likely reflect local habitat complexity, illustrating the flexibility of the Random Forest model in weighting features relevant to site-specific conditions.

### 3.3. Model Sensitivity, Overfitting Dynamics and Train-Test Thresholds

The final model configuration utilising the Growing season (2020) temporal window, the combined bands and indices feature set and an 80:20 train-test split serves as a reference baseline for all final performance metrics reported in this study. This configuration provided the most representative sampling method, yielding an accuracy of 95.24% and κ of 0.9389 for Scragh, an accuracy of 94.13% and κ of 0.9330 for Liskeenan and an accuracy of 94.43% and κ of 0.9348 for Ballymore. The κ for each site represents the degree of agreement between predicted and true classes, reflecting high internal pixel-level agreement under standard data splitting, though remaining subject to spatial autocorrelation as previously noted.

To evaluate the sensitivity of the models, the amount of training data for the model was systematically reduced from 80% to 20%. Results found that there is a noticeable decline in model performance as training data decreases across all sites (Table 7, Table 8 and Table 9). Training accuracy is presented alongside testing metrics to demonstrate the influence of varying split ratios on model convergence and potential overfitting. While training accuracy reflects how well the model memorises the training subset, the OA and κ coefficient derived from the test sets represent the model’s internal predictive performance within these spatial constraints.

Results demonstrate a non-linear decline in model predictive performance as training volume decreases. Performance holds steady until the 50:50 data split. As the training data is reduced beyond this level, the OA and κ decline due to a lack of data. At the same time, training accuracy increases. This highlights a case of model degradation where the classifier overfits to smaller training subsets, thereby hindering generalisability to unseen data.

From a resource-management perspective, reducing training data from an 80:20 to a 50:50 split resulted in minor accuracy decreases across a single partitioning experiment: 1.39% for Scragh, 1.60% for Liskeenan, and 1.15% for Ballymore. While these thresholds vary depending on local spatial autocorrelation and minority class distributions, this empirical result indicates that high within-site accuracy can be maintained with reduced reference sample volumes, provided key classes remain adequately represented.

### 3.4. Habitat Area Classification Evaluation

To assess the model’s reliability beyond accuracy metrics, classification maps and total areas for each habitat class were calculated for each site using the data splits analysed in the previous section. To visualise habitat classes, a site-specific colour scheme was implemented for each study site. Given the limitations of generating 20+ distinct colours to represent each class within GEE, each site was assigned a unique colour palette to maximise visual separability between habitat classes present at each site. This approach ensured that the output classification maps were not constrained by software limitations and remained representative of the actual site data.

**Figure 5:**
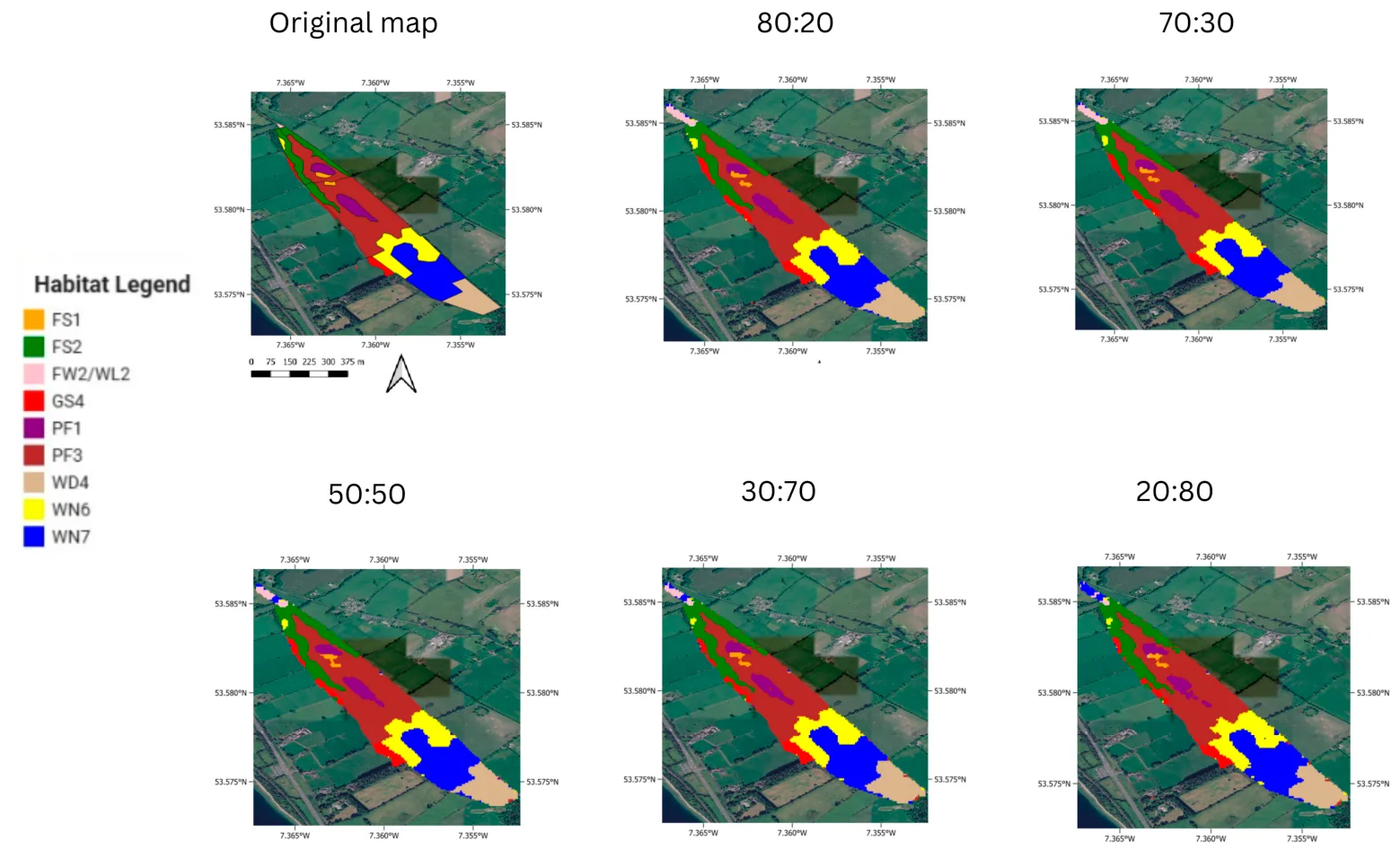
Comparison of original and classified habitat maps for Scragh. All panels share the same spatial extent, coordinate reference system and scale as shown in the top-left panel.

**Figure 6:**
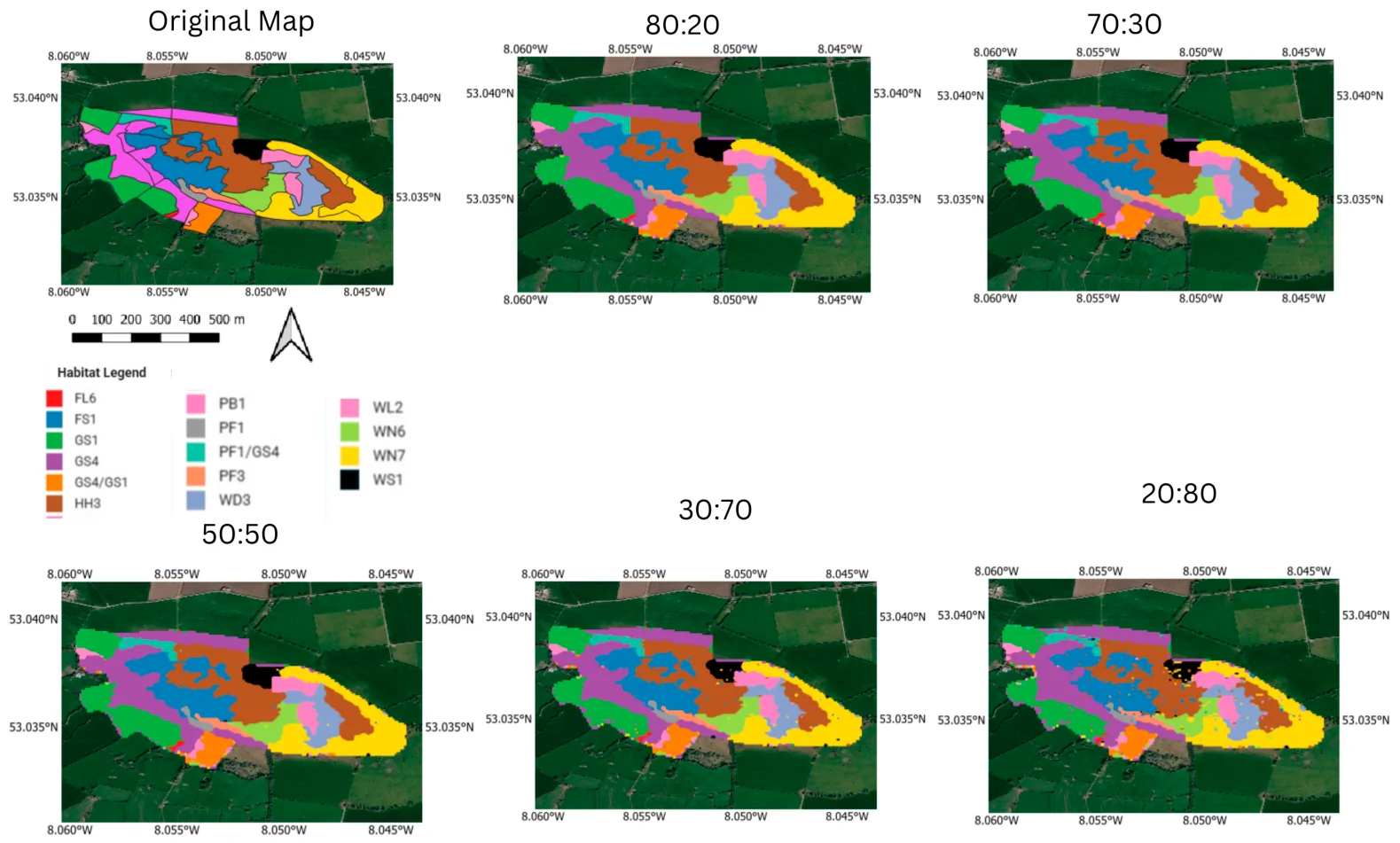
Comparison of original and classified habitat maps for Liskeenan. All panels share the same spatial extent, coordinate reference system and scale as shown in the top-left panel.

**Figure 7:**
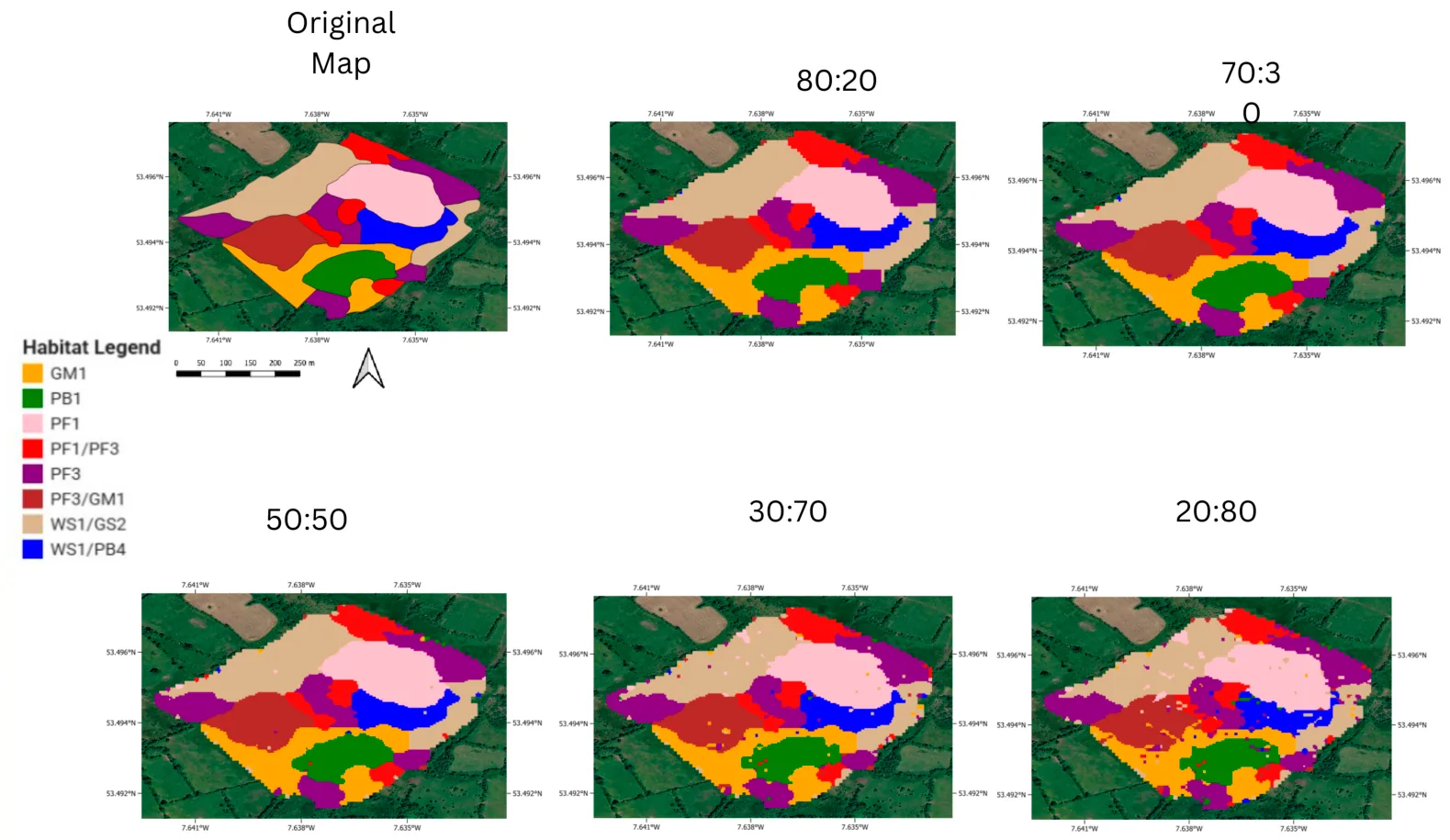
Comparison of original and classified habitat maps for Ballymore. All panels share the same spatial extent, coordinate reference system and scale as shown in the top-left panel.

As training data availability decreased, the resulting classification maps displayed an increase in salt-and-pepper noise, notably at class boundaries, though some noise was also noticed inside larger class polygons despite the application of the post-classification majority filter (Figure 5, Figure 6 and Figure 7). This suggests while the model retains its ability to identify dominant habitat classes, when the training data is reduced, the model’s ability to distinguish smaller classes decreases.

Ballymore shows the most salt-and-pepper noise due to the site’s complex habitat composition (Figure 7). Ballymore differs from the other two sites due to its mix of habitat classes. This inter-class fragmentation creates fuzzy boundaries and spectral confusion. In the mixed classes, habitat boundaries are indistinct, exhibiting a continuous transition between vegetation types. This challenges the classifier’s ability to distinguish between classes, frequently resulting in misclassifications as training data volume is reduced.

**Figure 8:**
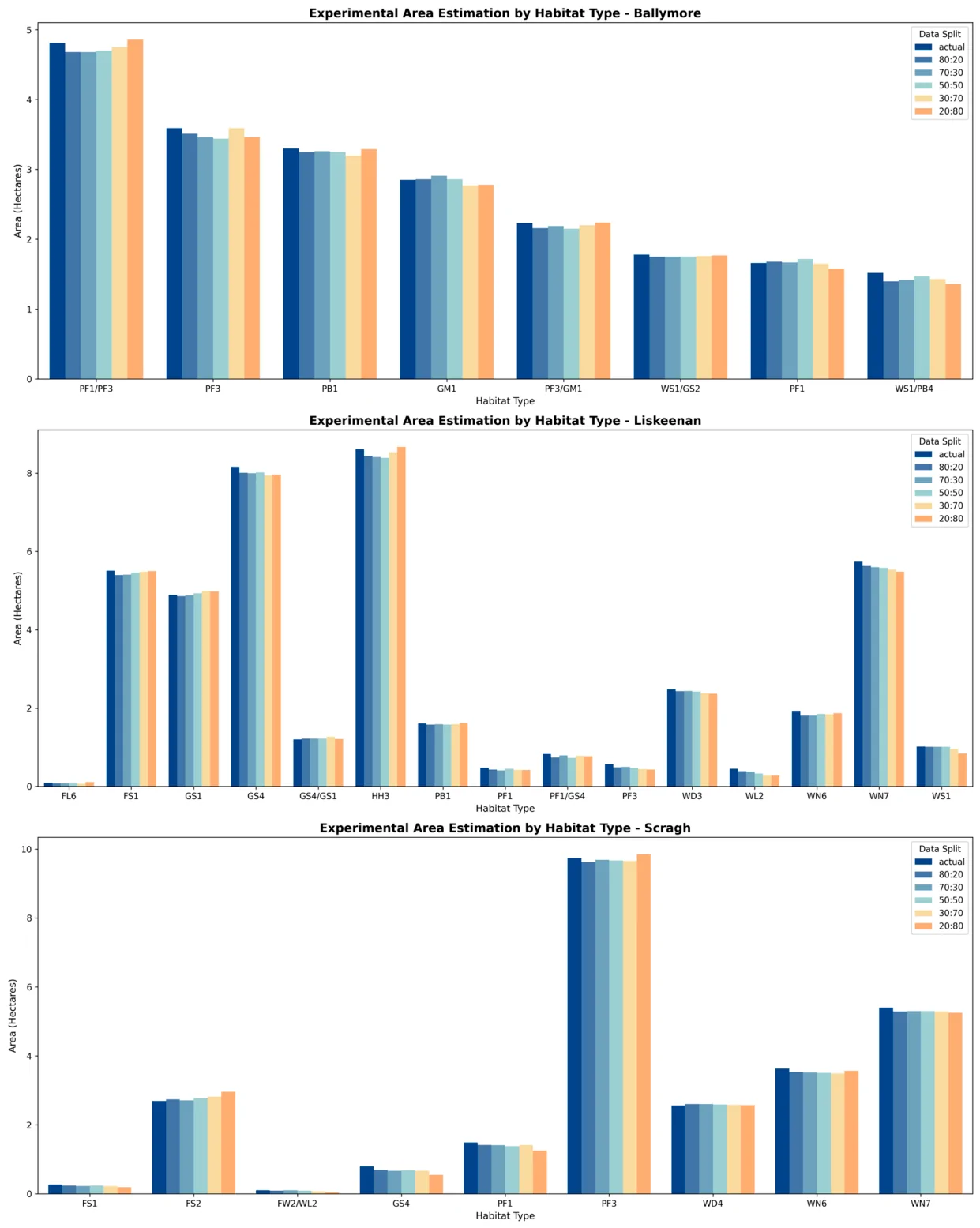
Observed and estimated habitat areas under different data splits.

Following the classification process, the total area for each habitat class was derived directly from the classified habitat maps. Figure 8 depicts the calculated habitat area in hectares for each study site, compared across the data splits.

Analysis of the classification maps and area outputs reveals a clear difference between model performance across habitat class size. Habitats with large spatial coverage exhibited high model stability due to greater sample availability. Although performance varied across smaller classes, broader land-cover types yielded consistently reliable results. However, minority classes are significantly more sensitive to training data reduction. As the amount of data reduces, the model struggles with rarer classes, leading to misclassification and large area estimation fluctuations. Detailed site-specific habitat area calculations in hectares are provided in Appendix A (Table A2, Table A3 and Table A4). Across all splits and sites, the total classified raster area exhibits a consistent, minor negative offset of approximately 2% relative to the original vector reference area (e.g., ~26.2 ha vs. 26.7 ha at Scragh; ~42.5 ha vs. 43.6 ha at Liskeenan; ~21.3 ha vs. 21.7 ha at Ballymore). This baseline area differential stems primarily from edge-pixel selection along the irregular perimeters of the site polygons, where boundary pixels are excluded if their centroids lie outside the vector boundaries during Google Earth Engine (GEE) rasterisation. Furthermore, post-classification 3 × 3 majority filtering lightly smooths fine-scale outer boundary pixels, accounting for the slight, uniform negative area errors reported in Appendix A (Table A5, Table A6 and Table A7).

Rare and highly localised classes exhibited higher relative error percentages, as minor pixel-level misclassifications and boundary effects disproportionately affect smaller spatial footprints. Specifically, class-level omission errors lead to the underestimation of mapped habitat extent, while commission errors result in overestimation.

Consequently, while the configured classification workflow effectively captures broader ecosystem composition and macro-scale structural changes, fine-scale spatial allocations for rare classes should be interpreted with consideration of these localised mapping constraints. To further evaluate the final classification model (growing season, combining indices and bands with an 80:20 split), per-class performance was assessed using Producer’s and User’s Accuracies as detailed in Table 10, Table 11 and Table 12.

**Figure A1:**
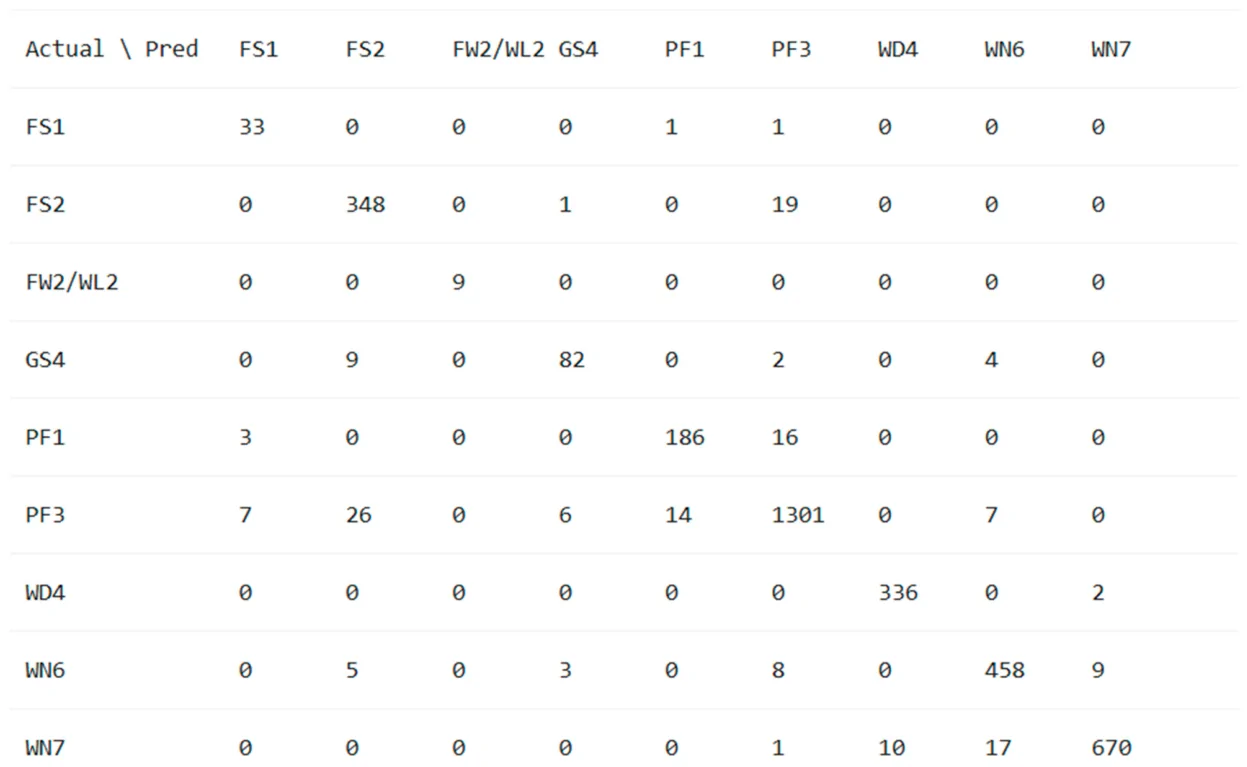
Confusion matrix for the final classification model at Scragh.

**Figure A2:**
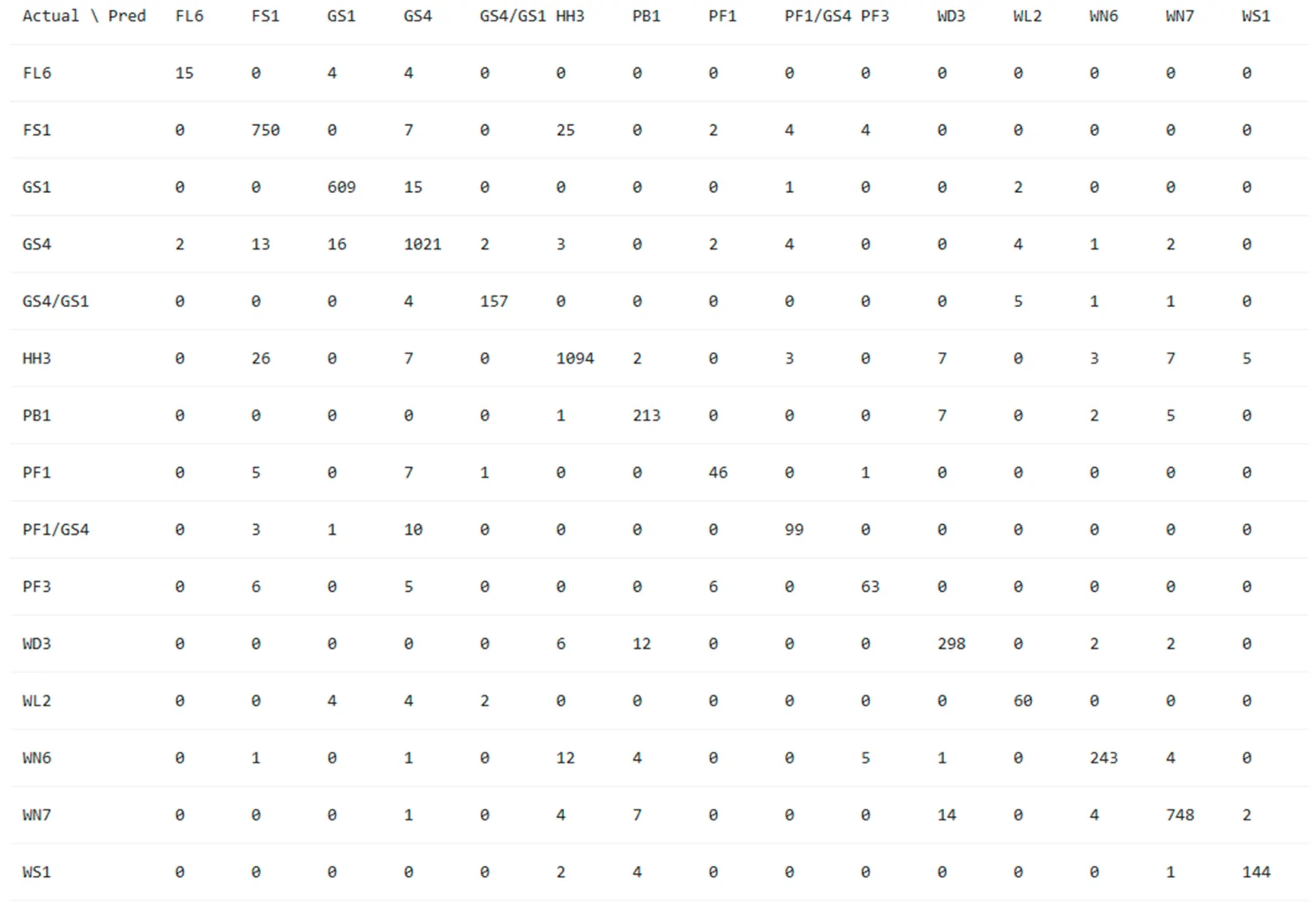
Confusion matrix for the final classification model at Liskeenan.

**Figure A3:**
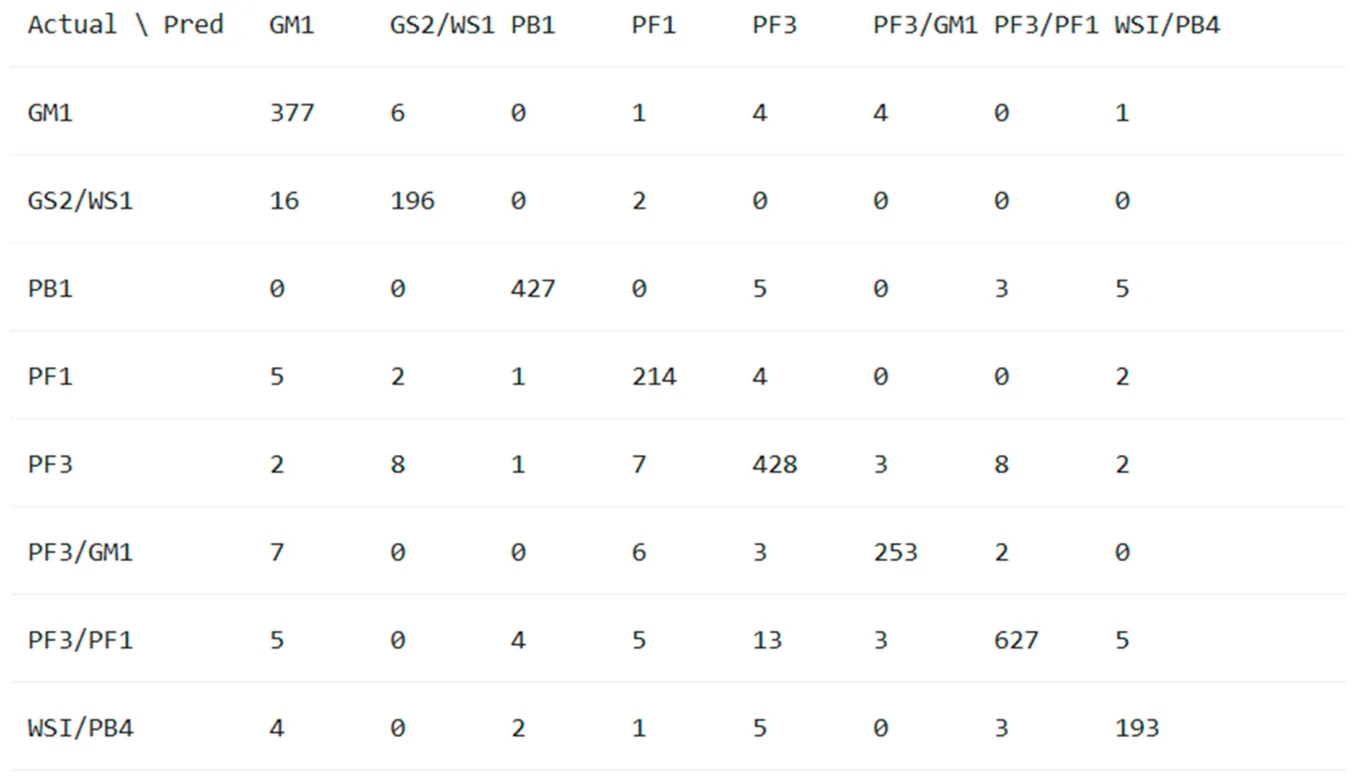
Confusion matrix for the final classification model at Ballymore.

As detailed in Table 10, Table 11 and Table 12, per-class performance varied across sites, revealing distinct trends for minority or transitional habitat types. While major classes consistently achieved high accuracies, rare or structurally complex classes experienced lower Producer’s Accuracy due to omission errors. Specifically, omission errors cause habitat patches to be missed (leading to area underestimation), whereas commission errors introduce false positives (resulting in area inflation). For instance, at Liskeenan (Table 11), rare or narrow transition classes recorded noticeably lower Producer’s Accuracies, such as FL6 (65.22%), PF1(76.67%) and PF3 (78.75%). Similarly, at Scragh (Table 10), FS1 showed lower User’s Accuracy at 76.74%. Where omission and commission errors fail to balance within a specific class, systemic bias emerges in final area totals. The full confusion matrices detailing the per-class User’s and Producer’s Accuracies for all sites can be found in the Appendix A (Figure A1, Figure A2 and Figure A3).

While F1-scores generally remain high and stable across well-represented classes, classes with small sample sizes occasionally produce skewed metrics. Conversely, densely sampled classes yield a stable and consistent model behaviour, emphasising the sensitivity of classification metrics to sample distribution.

## 4. Discussion

### 4.1. The Temporal Trade-Off: Phenological Windowing Versus Annual Noise

This research implemented various temporal windowing strategies to enhance land cover mapping. Specifically, it investigated how different timeframes affected overall model accuracy. Results showed that classification accuracies were generally consistent across all configurations, with a few notable exceptions. For Scragh, the results demonstrate a clear threshold in model performance driven by temporal data quality. While the band-only model maintains stable accuracy across temporal windows, both the indices-only and combined band-indices configurations exhibit some variation. The full-year windows using indices-only resulted in the lowest accuracy scores. Conversely, the Growing Season (2020) produced the maximum OA for combined features, suggesting that the minimisation of atmospheric interference is as vital as the feature set itself.

Regarding Liskeenan, results show no significant sensitivity to the inclusion of indices or seasonal constraints. The stability across full-year vs. growing season windows may indicate that the landscape features are spectrally distinct enough to be resolved regardless of the season. The reduced accuracy of the single scene approach highlights the limitations of relying on static temporal snapshots for habitat classification. This approach may not consistently achieve the same level of stability as a multi-temporal composition, as it frequently omits parts of the spectral phenological signature that aid in distinguishing between similar habitat types. Ballymore showed a similar pattern to the other two study sites, with high accuracies and little variation between full-year and growing season temporal windows. This suggests that the core spectral profiles of the primary habitat classes present at this site may possess sufficient descriptive variation to be distinguished across multiple temporal scales, though subtle transitional boundaries remain highly complex.

While the Wilcoxon test yielded no significant difference between the Low cloud cover (<30%) (2020) and Growing season (2020) medians, this result reinforces the limitations of relying solely on central tendency metrics in habitat classification. Prior studies have integrated statistical testing with box plots to effectively visualise and analyse data distribution [73,74]. The box plots for these two windows reveal that the distribution and spread of the data differ notably. In spectral analysis the model’s ability to classify depends more on inter-class separation than median values. The full-year data exhibit lower variance, which suggests that the inclusion of dormant-season noise suppresses the spectral signatures of different habitats into an overlapping, homogenised range. Even if the medians are similar, the full-year data confuses the decision boundaries making it difficult for the classifier to isolate specific features. This result is consistent with the temporal-window framework established by Van Niel and McVicar [75], that isolating specific growth phases maximised classification accuracy while reducing the spectral noise and data redundancy found within full-year composites. The choice of growing season is justified by the increased spectral contrast, variance and lower computational demand which is crucial for model convergence, regardless of the similarities in central tendency and accuracy. Building upon the framework established by Yi et al. [76], this study confirms that utilising images from active growth periods provides critical data for mapping and is essential for achieving peak classification accuracy and habitat detection.

The growing-season window was selected for the final classification workflow because it maximised spectral separability among habitat classes while reducing the influence of dormant-season noise [77,78]. By focusing on the period of active vegetation development, this temporal window reduced the influence of dormant-season noise. While utilising a full-year composite provides a wealth of data, the introduction of seasonal noise remains a risk; specifically, extreme weather events within a given year can distort the baseline data and skew future comparative research.

While conventional remote sensing research favours annual datasets [11,25,33], the results suggest that the growing season offers a viable alternative to full-year windowing for habitat classification in certain contexts. Crucially, this approach offers significant operational advantages: by narrowing the temporal window, it is possible to achieve reliable habitat mapping while simultaneously reducing the demand for computation resources and lowering data processing costs. This trade-off confirms that the temporal alignment of observations is just as important as the total data sample size. By isolating the growing season, it is possible to capture the peak phenological discriminability of the habitats, providing a streamlined framework that maintains high accuracy without the computational burden of larger annual datasets.

### 4.2. Performance of Synergistic Stacking

Following the selection of the growing season as the chosen temporal window, the analysis focused on determining the most effective feature set. The integration of spectral information is a critical element in increasing the accuracy as demonstrated by Kobayashi et al. and Bacaksiz [79,80]. While individual feature sets possess unique strengths, feature stacking generally yields superior classification results.

Across all study sites, models utilising spectral indices alone exhibited the lowest classification performance. Although effective at capturing biophysical traits, models lacking raw bands forfeit essential radiometric information critical to classification [81]. This compresses the spectral profile and limits the model to pre-determined biophysical relationships.

The combined feature stack produced the peak OA at only one site (Scragh, 95.24%), whereas maximum class separation at Liskeenan and Ballymore was achieved using raw spectral bands alone. These site-dependent results indicate that including auxiliary spectral indices can introduce feature redundancy or collinear noise that slightly degrades localised model performance. However, this accuracy trade-off was minimal amounting to a minor 0.24% difference at Liskeenan and 1.65% at Ballymore.

Relying solely on raw reflectance data carries a high risk of site-level overfitting, as individual bands are inherently susceptible to local soil background noise, illumination geometry, and atmospheric variance. In contrast, normalised spectral indices (e.g., EVI, SAVI, and NDWI) provide essential biophysical context while mathematically dampening background noise [82]. Although site-specific feature selection can yield marginal performance gains, adopting customised subsets introduces unnecessary operational complexity and hinders automated deployment. Therefore, the combined feature set was retained as a standardised workflow, ensuring a unified, transferable methodology that maintains high average accuracy while optimising operational simplicity for regional-scale wetland mapping.

Across all three sites a mix of indices and spectral populated the top qualitative relative rankings, providing a rich feature space that allows the model to leverage both raw spectral reflectance and biophysical information. While variable importance metrics confirm the strong utility of these predictors, many spectral bands and indices naturally exhibit high collinearity. Consequently, feature importance rankings reflect qualitative relative preferences among correlated variables rather than isolated, independent contributions. This pattern is clearly observed in the lower-ranking features across all three sites in Figure 4, where NDVI, RVI, NRVI, and SAVI share almost identical, subtle importance profiles.

Furthermore, given that the raw reflectance values operate in the thousands without scaling, the soil-adjustment factor (L = 0.5) within SAVI has a minimal mathematical impact, yielding behaviour virtually identical to NDVI. As a result, the Random Forest model divides importance lightly across this cluster of collinear indices, allowing distinct formulations like EVI and GRVI to effectively highlight unique data variance. Our feature configuration intentionally prioritised visible and near-infrared metrics widely validated in wetland remote sensing, ensuring a streamlined, robust feature stack that enhances model stability and performance [37,83,84].

### 4.3. Model Evaluation: Comparison of Train-Test Split Ratios and Sampling Strategies

Traditionally, ML models require large volumes of data often utilising a data split of 70:30/80:20 [85,86,87]. The relatively stable performance observed at the 50:50 split indicates that, within this specific partitioning experiment, the Random Forest model maintained strong within-site accuracy despite reduced reference data volume.

However, further reductions to the 30:70 and 20:80 splits resulted in pronounced performance degradation and increased sensitivity to sample scarcity, particularly for minority habitat classes. By showing that a 50:50 split remains viable, this study highlights a manageable tolerance for smaller training sets, supporting ecological monitoring efforts where resource limitations constrain extensive field data collection.

The success of this reduced training data volume is not synonymous with all data selection methods; rather, it is contingent upon the specific selection process employed. For instance, Shi et al. [88] analysed in hyperspectral image classification how different query strategies selected the most informative data points. Xu et al. [89] states that while feature selection can reduce the volume of data, selection should focus on keeping the underlying data information such as geometry and gradient. Obtaining high classification accuracy with limited data is heavily reliant upon a stratified approach to sample selection. For the RF model, it is advantageous if training pixels are strategically distributed to capture the core spectral signature of each distinct class. If sampling is clustered or neglects transitional habitats, the risk of model overfitting increases significantly [90,91].

Moving towards a 50:50 data split shifts large-scale data collection to targeted field sampling. This targeted approach produces information-dense data. The remaining half of the site then acts as an internal within-site test set, acknowledging the influence of spatial autocorrelation.

The 50:50 partition demonstrates high empirical efficacy for these specific habitat configurations; however, because these findings are derived from a single random partition utilising GEE default seed, they retain some sensitivity to this specific allocation. This outcome underscores that suitable sample reduction thresholds are inherently dataset-dependent rather than universally applicable in remote sensing mapping.

While total area calculations remained fairly consistent even with the most extreme data splits, these extreme splits revealed clear limitations in class-specific area estimations. The reduction in training data occasionally resulted in the loss of minor habitat areas, which were subsequently absorbed into larger classes.

### 4.4. Scalability of 5 m Sentinel-2 Resampling and Workflow Transferability

While the Sentinel-2 constellation operates natively at a 10 m spatial resolution [83], resampling this data to a 5 m grid was implemented strictly to improve geometric alignment and boundary representation within the reference polygons, rather than to enhance the native spatial resolution of the sensor. Operating at this 5 m sampling interval provided a practical balance for aligning fragmented reference polygons with Sentinel-2-derived spectral information while limiting excessive pixel-level noise [92], representing a recognised approach in remote sensing. To gather spectral data at this selected scale the GEE sampleRegions() function was used [93]. This extracts the pixel-level values directly from the resampled 5 m grid at each specified vector coordinate using nearest-neighbour interpolation without generating true physical super-resolution. In addition, running sampleRegions() within GEE reduces the substantial storage and processing burden commonly associated with high-resolution datasets, ensuring a highly scalable and reproducible workflow [94,95,96].

While localised pixel-level splits are subject to spatial autocorrelation, the consistency of the classification metrics across all three independent study sites suggests that the developed methodology indicates within-site robustness and workflow reproducibility. The RF model exhibited consistency between accuracy metrics across all sites. Scragh achieved an OA of 95.24% (k = 0.9389). This performance was mirrored in Liskeenan OA = 94.13% (k = 0.9330) and Ballymore OA = 94.43% (k = 0.9348). While OA exceeding 90% is high for complex wetland ecosystems, this performance likely reflects the tailored workflow. The narrow range of accuracies suggests internal methodological consistency and stability within the tested fen environments. While this internal consistency highlights the feasibility of the selected combination of 5 m resampled Sentinel-2 imagery and temporal windowing within the study sites, applying this framework directly to entirely new fen ecosystems without localised data might require further validation.

### 4.5. Practical Implications, Model Limitations, and Future Directions

The site-level performance demonstrated by this workflow offers a viable alternative to traditional on-site mapping constraints, thereby minimising field labour allocation, mitigating high operational costs and optimising the utilisation of specialised ecological expertise.

Crucially, the results suggest that the stability of the RF model with only 50% training data could enable a significant shift in field data collection strategies. Field campaigns can consequently transition from broad, exhaustive ecosystem mapping to collecting small, highly targeted habitat datasets. This approach may allow a larger proportion of labelled reference data to be reserved for internal within-site testing, while independent validation still requires spatially separate reference data. By efficiently leveraging limited on-site resources, this streamlined approach ensures that ecological expertise is focused on high-value inputs rather than redundant data collection. This enables ecological teams to scale their monitoring across much larger geographical areas.

However, employing this downsized approach introduces specific trade-offs that must be considered. As previously mentioned, under more restrictive splits, rarer habitat classes risk being lost by the classifier because training data is constrained and minor classes may be consumed by more dominant classes. Ultimately, reducing data volume without sacrificing model accuracy requires strict, expert-driven classification of training data to ensure small-scale habitats are included in the final maps.

Furthermore, several technical limitations must be considered. Due to the inherent mixed pixel effect found in Sentinel-2, post-classification majority filtering may result in narrow features and fragmented habitat classes being absorbed by dominant surrounding land cover. Atmospheric interference such as severe cloud cover limits the availability of pristine imagery within the temporal windows. Finally, while the RF algorithm provided a highly accurate framework, it lacks the capacity to capture the complex spatial context and structural texture that advanced deep learning models exploit. Future research should explore a shift from purely spectral models toward hybrid approaches—integrating spatial-spectral deep learning or object-based image analysis (OBIA)—to better capture the intricate structural boundaries characteristic of vulnerable fen ecosystems.

Topographic data, such as the Shuttle Radar Topography Mission (SRTM), were excluded due to spatial resolution mismatches and the characteristically flat terrain of Irish fens, where SRTM’s coarse elevation metrics cannot capture fine-scale habitat variations. Similarly, this purely optical framework omits soil moisture data.

To refine the framework, future research should incorporate hydrological modelling alongside high-resolution LiDAR data to capture microtopographic variations. Additionally, future iterations will evaluate model stability across multiple random seeds to systematically quantify variability and address sample partitioning dependencies. Finally, incorporating explicit pixel-level cloud and shadow masking algorithms (e.g., QA60 or SCL-based filtering) will ensure tighter quality control over input temporal composites.

## 5. Conclusions

This study demonstrates a reproducible remote sensing workflow within GEE to map complex fen ecosystems across the investigated study sites. The methodology systematically analysed the influence of temporal windowing, feature sets, and training data splits on classification accuracy and model performance. Restricting the temporal window to a summer period (June-September) captured peak phenological differences among habitats, showing that targeted seasonal imagery provides a clearer classification signal than broad annual composites affected by temporal noise. The targeted approach yielded within-site classification accuracies of 95.24% at Scragh, 94.13% at Liskeenan and 94.43% at Ballymore.

The combination of raw spectral bands and targeted vegetation indices provided the necessary spectral separability to distinguish similar habitat classes across all three landscapes. The combined feature stack performed best at Scragh (95.24%), whereas at Liskeenan and Ballymore, utilising raw bands alone yielded the highest accuracies (94.37% and 96.08%, respectively). However, at Liskeenan, the accuracy advantage of raw bands over the combined stack was negligible (0.24%), indicating functional parity between the configurations. These subtle variations suggest that feature utility is somewhat site-dependent: at Ballymore, additional indices may have introduced minor feature redundancy in complex, mixed habitats, whereas at Scragh, the expanded feature space provided crucial complementary information.

Nevertheless, the combined feature set was selected as the final standard configuration because it yields a stable performance baseline across all three sites. Furthermore, adopting a single operational pipeline eliminates the need for site-specific feature engineering, ensuring the workflow remains streamlined, reproducible, and easily automated for regional-scale wetland mapping.

Evaluation of varying train-test split ratios indicated that a 50:50 split provided an effective empirical compromise between training sample size and reliable validation performance. At the 50:50 split, Scragh resulted in κ of 0.9217, OA of 93.85%. Liskeenan had a κ of 0.9142, OA of 92.53%. Similarly, Ballymore exhibited a κ of 0.9217, OA value of 93.28%. When the training data availability was further reduced, performance degraded due to sample scarcity, particularly impacting rare and fragmented habitat classes that were occasionally misclassified and absorbed into surrounding larger classes. While a 50:50 split served as an effective empirical compromise, multi-seed validation is recommended for broader application to account for sampling variance.

In conclusion, this research evaluated the operational viability of leveraging GEE-based satellite imagery for Irish fen mapping and monitoring. Although the proposed workflow achieves consistent within-site reproducibility, direct transferability to novel geographic regions remains contingent on the availability of localised reference data.

These findings directly address the study’s core objectives. Reducing the volume of required training data minimises on-site mapping demands, enabling conservation organisations to cut field labor costs and scale ecological monitoring across much broader areas. Although training data requirements can be reduced, an independent, dedicated subset of field data remains essential to serve as an uncompromised validation baseline—a necessity that grows increasingly critical amid a shrinking ecological workforce.

## Figures and Tables

**Table 1:** Temporal windowing configurations evaluated.

Window	Description	No. of Bands	No. of Auxiliary Variables	No. of Scenes per Site Scragh/Liskeenan/Ballymore
**1**	Full year (2019)	4	8	33/40/38
**2**	Full year (2020)	4	8	39/43/45
**3**	Low cloud cover (<30%) (2020)	4	8	21/21/20
**4**	Growing season (2020)	4	8	5/4/4
**5**	Best single scene per site with lowest cloud cover (2019–2020)	4	8	1/1/1

**Table 2:** Summary of Sentinel-2 spectral bands and auxiliary indices used in the classification.

Category	Variables	Description	Reference
**Colour image bands**	Blue (B2)	Spectral reflectance value for the blue band	
	Green (B3)	Spectral reflectance value for the green band	
	Red (B4)	Spectral reflectance value for the red band	
	Near-infrared (NIR) (B8)	Spectral reflectance value for the NIR band	
**Spectral indices**	Normalised difference Vegetation Index (NDVI)	$\frac{\left(NIR-RED\right)}{\left(NIR+RED\right)}$	[1]
	Ratio Vegetation Index (RVI)	$\frac{NIR}{RED}$	[54]
	Soil Adjusted Vegetation Index (SAVI)	$\frac{\left(NIR-RED\right)}{\left(NIR+RED+L\right)}\times \left(1+L\right)$	[55]
	Enhanced Vegetation Index (EVI)	$2.5\left(\frac{\left(NIR-RED\right)}{NIR+6\cdot RED-7.5BLUE+1}\right)$	[56]
	Green normalised difference vegetation Index (GNDVI)	$\frac{\left(NIR-GREEN\right)}{\left(NIR+GREEN\right)}$	[20]
	Green-red Vegetation Index (GRVI)	$\frac{\left(GREEN-RED\right)}{\left(GREEN+RED\right)}$	[57]
	Normalised Ratio Vegetation Index (NRVI)	$\frac{\left(RVI-1\right)}{\left(RVI+1\right)}$	[53]
	Normalised difference Water Index (NDWI)	$\frac{\left(GREEN-NIR\right)}{\left(GREEN\;+\;NIR\right)}$	[58]

**Table 3:** Overall Accuracy (%) from the classification results for Scragh.

Temporal Window	Bands Only	Indices Only	Bands and Indices
**Full year (2019)**	94.76	64.02	80.93
**Full year (2020)**	94.73	68.78	83.93
**Low cloud cover (<30%) (2020)**	94.77	95.07	94.83
**Growing season (2020)**	95.15	94.80	95.24
**Best single scene per site with lowest cloud cover (2019–2020)**	94.03	93.92	94.69

**Table 4:** Overall Accuracy (%) from the classification results for Liskeenan.

Temporal Window	Bands Only	Indices Only	Bands and Indices
**Full year (2019)**	94.07	93.87	94.06
**Full year (2020)**	94.17	93.98	94.14
**Low cloud cover (<30%) (2020)**	94.05	94.11	93.87
**Growing season (2020)**	94.37	94.09	94.13
**Best single scene per site with lowest cloud cover (2019–2020)**	93.70	92.80	92.89

**Table 5:** Overall Accuracy (%) from the classification results for Ballymore.

Temporal Window	Bands Only	Indices Only	Bands and Indices
**Full year (2019)**	97.13	96.19	96.18
**Full year (2020)**	95.85	96.74	97.12
**Low cloud cover (<30%) (2020)**	96.66	97.02	96.43
**Growing season (2020)**	96.08	95.95	94.43
**Best single scene per site with lowest cloud cover (2019–2020)**	94.87	94.47	95.15

**Table 6:** Overall Accuracy (%) for feature sets by site.

Site	Feature Set	Overall Accuracy (%)
**Scragh**	Bands	95.15
	Indices	94.80
	Bands & Indices	95.24
**Liskeenan**	Bands	94.37
	Indices	94.09
	Bands & Indices	94.13
**Ballymore**	Bands	96.08
	Indices	95.95
	Bands & Indices	94.43

**Table 7:** Performance metrics across training and testing splits for Scragh.

Train-Test Split	Kappa Coefficient	Overall Accuracy (%)	Training Accuracy (%)
**80:20**	0.9389	95.24	96.52
**70:30**	0.9349	94.94	96.66
**50:50**	0.9217	93.85	96.77
**30:70**	0.8822	90.81	97.32
**20:80**	0.8212	86.14	97.61

**Table 8:** Performance metrics across training and testing splits for Liskeenan.

Train-Test Split	Kappa Coefficient	Overall Accuracy (%)	Training Accuracy (%)
**80:20**	0.9330	94.13	96.02
**70:30**	0.9235	93.60	96.20
**50:50**	0.9142	92.53	96.57
**30:70**	0.8567	87.55	97.21
**20:80**	0.7765	80.58	97.54

**Table 9:** Performance metrics across training and testing splits for Ballymore.

Train-Test Split	Kappa Coefficient	Overall Accuracy (%)	Training Accuracy (%)
**80:20**	0.9348	94.43	96.23
**70:30**	0.9308	94.07	96.45
**50:50**	0.9217	93.28	96.99
**30:70**	0.8867	90.33	97.33
**20:80**	0.8240	84.94	98.00

**Table 10:** Per-class performance for the final classification model at Scragh.

Habitat Code	Producer’s Accuracy (%)	User’s Accuracy (%)	F1-Score (%)	Reference Samples per Class
**FS1**	94.29	76.74	84.62	35
**FS2**	94.57	89.69	92.06	368
**FW2/WL2**	100	100	100	9
**GS4**	84.54	89.13	86.77	97
**PF1**	90.73	92.54	91.63	205
**PF3**	95.59	96.51	96.05	1361
**WD4**	99.41	97.11	98.25	338
**WN6**	94.82	94.24	94.53	483
**WN7**	95.99	98.38	97.17	698

**Table 11:** Per-class performance for the final classification model at Liskeenan.

Habitat Code	Producer’s Accuracy (%)	User’s Accuracy (%)	F1-Score (%)	Reference Samples per Class
**FL6**	65.22	88.24	75.00	23
**FS1**	94.70	93.28	93.98	792
**GS1**	97.13	96.06	96.59	627
**GS4**	95.42	94.01	94.71	1070
**GS4/GS1**	93.45	96.91	95.15	168
**HH3**	94.80	95.38	95.09	1154
**PB1**	93.42	88.02	90.64	228
**PF1**	76.67	82.14	79.31	60
**PF1/GS4**	87.61	89.19	88.39	113
**PF3**	78.75	86.30	82.35	80
**WD3**	93.13	91.13	92.12	320
**WL2**	85.71	84.51	85.11	70
**WN6**	89.67	94.92	92.22	271
**WN7**	95.90	97.14	96.52	780
**WS1**	95.36	95.36	95.36	151

**Table 12:** Per-class performance for the final classification model at Ballymore.

Habitat Code	Producer’s Accuracy (%)	User’s Accuracy (%)	F1-Score (%)	Reference Samples per Class
**GM1**	95.93	90.63	93.20	393
**WS1/GS2**	91.59	92.45	92.02	214
**PB1**	97.05	98.16	97.60	440
**PF1**	93.86	90.68	92.24	228
**PF3**	93.25	92.64	92.94	459
**PF3/GM1**	93.36	96.20	94.76	271
**PF3/PF1**	94.71	97.51	96.09	662
**WS1/PB4**	92.79	92.79	92.79	208

## Data Availability

The Pilot Fen Survey 2019–2020 data analysed during the current study were provided by the National Parks and Wildlife Service (NPWS) under license/permission and are not publicly available. Requests to access these datasets should be directed to the NPWS at NPWS_Data@npws.gov.ie. The multiple scripts created and utilised in this study (implemented via Google Earth Engine API) can all be viewed in our GitHub repository (v1.0) https://github.com/CiaraFarrellSETU/Optimising-Irish-Fen-Habitat-Mapping-Codes (accessed on 13 October 2026).

## Abbreviations

The following abbreviations are used in this manuscript:

RS	Remote Sensing
GEE	Google Earth Engine
ML	Machine Learning
RF	Random Forest
SAC	Special Areas of Conservation
BOA	Bottom of Atmosphere
NPWS	National Parks and Wildlife Service
NIR	Near-infrared
NDVI	Normalised Difference Vegetation Index
RVI	Ratio Vegetation Index
SAVI	Soil Adjusted Vegetation Index
EVI	Enhanced Vegetation Index
GNDVI	Green normalised difference vegetation Index
GRVI	Green-red Vegetation Index
NRVI	Normalised Ratio Vegetation Index
NDWI	Normalised difference Water Index
κ	Kappa
OA	Overall Accuracy

## Appendix A

**Table A1:** Comparative Inventory of Identified Fossitt Habitats within the Study Sites.

Fossitt Codes	Official Habitat Description	Scragh Bog	Liskeenan Fen	Ballymore Fen	Polygon Count per Habitat
**FL6**	Turloughs		✓		1
**FS1**	Reed and large sedge swamps	✓	✓		4
**FS2**	Tall-herb swamps	✓			3
**FW2/WL2**	Depositing/lowland rivers & treelines	✓			1
**GM1**	Marsh			✓	2
**GS1**	Dry calcareous and neutral grassland		✓		3
**WS1/GS2**	Dry Meadows and Grassy Verges/Scrub			✓	1
**GS4**	Wet grassland	✓	✓		8
**GS4/GS1**	Wet grassland/Dry calcareous and neutral grassland		✓		1
**HH3**	Wet heath		✓		2
**PB1**	Raised bog		✓	✓	3
**PF1**	Rich fen and flush	✓	✓	✓	7
**PF1/GS4**	Rich fen and flush/Wet grassland		✓		1
**PF3**	Transition mire and quaking bog	✓	✓	✓	7
**PF3/GM1**	Transition mire and quaking bog/Marsh			✓	1
**PF3/PF1**	Transition mire and quaking bog/Rich fen and flush			✓	3
**WD3**	(Mixed) conifer woodland		✓		1
**WD4**	Conifer plantation	✓			1
**WL2**	Treelines		✓		2
**WN6**	Wet willow-alder-ash woodland	✓	✓		3
**WN7**	Bog woodland	✓	✓		5
**WS1**	Scrub		✓		1
**WS1/GS2**	Scrub/Cutover bog			✓	1

Note: A checkmark (✓) indicates the presence of the identified Fossitt habitat within the respective study site.

**Table A2:** Habitat Area Calculation for Scragh Bog (ha).

	FS1	FS2	FW2/WL2	GS4	PF1	PF3	WD4	WN6	WN7
**Actual Area**	0.27	2.69	0.10	0.79	1.49	9.74	2.56	3.63	5.40
**80:20**	0.24	2.74	0.09	0.69	1.42	9.62	2.60	3.53	5.29
**70:30**	0.23	2.71	0.10	0.67	1.41	9.69	2.60	3.52	5.30
**50:50**	0.24	2.77	0.09	0.68	1.38	9.67	2.59	3.51	5.30
**30:70**	0.22	2.82	0.07	0.67	1.42	9.66	2.58	3.49	5.29
**20:80**	0.19	2.96	0.04	0.55	1.25	9.85	2.57	3.57	5.25

**Table A3:** Habitat Area Calculation for Liskeenan (ha).

	FL6	FS1	GS1	GS4	GS4/GS1	HH3	PB1	PF1	PF1/GS4	PF3	WD3	WL2	WN6	WN7	WS1
**Actual Area**	0.09	5.51	4.89	8.16	1.20	8.61	1.61	0.48	0.83	0.57	2.48	0.45	1.93	5.74	1.02
**80:20**	0.08	5.40	4.86	8.01	1.22	8.44	1.58	0.43	0.74	0.49	2.43	0.39	1.81	5.63	1.01
**70:30**	0.08	5.41	4.88	8.00	1.22	8.41	1.59	0.41	0.79	0.50	2.44	0.38	1.81	5.60	1.01
**50:50**	0.08	5.46	4.93	8.02	1.22	8.39	1.58	0.45	0.73	0.47	2.42	0.33	1.85	5.58	1.01
**30:70**	0.07	5.48	4.99	7.94	1.27	8.53	1.59	0.42	0.78	0.44	2.38	0.28	1.84	5.54	0.96
**20:80**	0.11	5.50	4.98	7.96	1.21	8.67	1.62	0.42	0.77	0.43	2.37	0.28	1.87	5.48	0.84

**Table A4:** Habitat Area Calculation for Ballymore (ha).

	PF3/PF1	PF3	PB1	GM1	PF3/GM1	WS1/GS2	PF1	WS1/PB4
**Actual Area**	4.81	3.59	3.30	2.85	2.23	1.78	1.66	1.52
**80:20**	4.68	3.51	3.25	2.86	2.16	1.75	1.68	1.40
**70:30**	4.68	3.46	3.26	2.91	2.19	1.75	1.67	1.42
**50:50**	4.70	3.44	3.25	2.86	2.15	1.75	1.72	1.47
**30:70**	4.75	3.59	3.20	2.77	2.20	1.76	1.65	1.43
**20:80**	4.86	3.46	3.29	2.78	2.24	1.77	1.58	1.36

**Table A5:** Absolute and relative area errors for each habitat class Scragh.

Habitat Class	Split Ratio	Absolute Error (ha)	Relative Error (%)
**FS1**	80:20	−0.03	−11.10%
	70:30	−0.04	−14.80%
	50:50	−0.03	−11.10%
	30:70	−0.05	−18.50%
	20:80	−0.08	−29.60%
**FS2**	80:20	+0.05	+1.9%
	70:30	+0.02	+0.7%
	50:50	+0.08	+3.0%
	30:70	+0.13	+4.8%
	20:80	+0.27	+10.0%
**FW2/WL2**	80:20	−0.01	−10.00%
	70:30	0	0.00%
	50:50	−0.01	−10.00%
	30:70	−0.03	−30.00%
	20:80	−0.06	−60.00%
**GS4**	80:20	−0.1	−12.70%
	70:30	−0.12	−15.20%
	50:50	−0.11	−13.90%
	30:70	−0.12	−15.20%
	20:80	−0.24	−30.40%
**PF1**	80:20	−0.07	−4.70%
	70:30	−0.08	−5.40%
	50:50	−0.11	−7.40%
	30:70	−0.07	−4.70%
	20:80	−0.24	−16.10%
**PF3**	80:20	−0.12	−1.20%
	70:30	−0.05	−0.50%
	50:50	−0.07	−0.70%
	30:70	−0.08	−0.80%
	20:80	+0.11	+1.1%
**WD4**	80:20	+0.04	+1.6%
	70:30	+0.04	+1.6%
	50:50	+0.03	+1.2%
	30:70	+0.02	+0.8%
	20:80	+0.01	+0.4%
**WN6**	80:20	−0.1	−2.80%
	70:30	−0.11	−3.00%
	50:50	−0.12	−3.30%
	30:70	−0.14	−3.90%
	20:80	−0.06	−1.60%
**WN7**	80:20	−0.11	−2.00%
	70:30	−0.1	−1.90%
	50:50	−0.1	−1.90%
	30:70	−0.11	−2.00%
	20:80	−0.15	−2.80%

**Table A6:** Absolute and relative area errors for each habitat class Liskeenan.

Habitat Class	Split Ratio	Absolute Error (ha)	Relative Error (%)
**FL6**	80:20	−0.01	−11.10%
	70:30	−0.01	−11.10%
	50:50	−0.01	−11.10%
	30:70	−0.02	−22.20%
	20:80	+0.02	+22.2%
**FS1**	80:20	−0.11	−2.00%
	70:30	−0.1	−1.80%
	50:50	−0.05	−0.90%
	30:70	−0.03	−0.50%
	20:80	−0.01	−0.20%
**GS1**	80:20	−0.03	−0.60%
	70:30	−0.01	−0.20%
	50:50	+0.04	+0.8%
	30:70	+0.10	+2.0%
	20:80	+0.09	+1.8%
**GS4**	80:20	−0.15	−1.80%
	70:30	−0.16	−2.00%
	50:50	−0.14	−1.70%
	30:70	−0.22	−2.70%
	20:80	−0.2	−2.50%
**GS4/GS1**	80:20	+0.02	+1.7%
	70:30	−0.02	−1.7%
	50:50	+0.02	+1.7%
	30:70	+0.07	+5.8%
	20:80	+0.01	+0.8%
**HH3**	80:20	−0.17	−2.00%
	70:30	−0.2	−2.30%
	50:50	−0.22	−2.60%
	30:70	−0.08	−0.90%
	20:80	+0.06	+0.7%
**PB1**	80:20	−0.03	−1.90%
	70:30	−0.02	−1.20%
	50:50	−0.03	−1.90%
	30:70	−0.02	−1.20%
	20:80	+0.01	+0.6%
**PF1**	80:20	−0.05	−10.40%
	70:30	−0.07	−14.60%
	50:50	−0.03	−6.30%
	30:70	−0.06	−12.50%
	20:80	−0.06	−12.50%
**PF1/GS4**	80:20	−0.09	−10.80%
	70:30	−0.04	−4.80%
	50:50	−0.1	−12.00%
	30:70	−0.05	−6.00%
	20:80	−0.06	−7.20%
**PF3**	80:20	−0.08	−14.00%
	70:30	−0.07	−12.30%
	50:50	−0.1	−17.50%
	30:70	−0.13	−22.80%
	20:80	−0.14	−24.60%
**WD3**	80:20	−0.05	−2.00%
	70:30	−0.04	−1.60%
	50:50	−0.06	−2.40%
	30:70	−0.1	−4.00%
	20:80	−0.11	−4.40%
**WL2**	80:20	−0.06	−13.30%
	70:30	−0.07	−15.60%
	50:50	−0.12	−26.70%
	30:70	−0.17	−37.80%
	20:80	−0.17	−37.80%
**WN6**	80:20	−0.12	−6.20%
	70:30	−0.12	−6.20%
	50:50	−0.08	−4.10%
	30:70	−0.09	−4.70%
	20:80	−0.06	−3.10%
**WN7**	80:20	−0.11	−1.90%
	70:30	−0.14	−2.40%
	50:50	−0.16	−2.80%
	30:70	−0.2	−3.50%
	20:80	−0.26	−4.50%
**WS1**	80:20	−0.01	−1.00%
	70:30	−0.01	−1.00%
	50:50	−0.01	−1.00%
	30:70	−0.06	−5.90%
	20:80	−0.18	−17.65%

**Table A7:** Absolute and relative area errors for each habitat class Ballymore.

Habitat Class	Split Ratio	Absolute Error (ha)	Relative Error (%)
**PF3/PF1**	80:20	−0.13	−2.70%
	70:30	−0.13	−2.70%
	50:50	−0.11	−2.30%
	30:70	−0.06	−1.20%
	20:80	+0.05	+1.0%
**PF3**	80:20	−0.08	−2.20%
	70:30	−0.13	−3.60%
	50:50	−0.15	−4.20%
	30:70	0	0.00%
	20:80	−0.13	−3.60%
**PB1**	80:20	−0.05	−1.50%
	70:30	−0.04	−1.20%
	50:50	−0.05	−1.50%
	30:70	−0.1	−3.00%
	20:80	−0.01	−0.30%
**GM1**	80:20	+0.01	+0.4%
	70:30	+0.06	+2.1%
	50:50	+0.01	+0.4%
	30:70	−0.08	−2.80%
	20:80	−0.07	−2.50%
**PF3/GM1**	80:20	−0.07	−3.10%
	70:30	−0.04	−1.80%
	50:50	−0.08	−3.60%
	30:70	−0.03	−1.30%
	20:80	+0.01	+0.4%
**WS1/GS2**	80:20	−0.03	−1.70%
	70:30	−0.03	−1.70%
	50:50	−0.03	−1.70%
	30:70	−0.02	−1.10%
	20:80	−0.01	−0.60%
**PF1**	80:20	+0.02	+1.2%
	70:30	+0.01	+0.6%
	50:50	+0.06	+3.6%
	30:70	−0.01	−0.60%
	20:80	−0.08	−4.80%
**WS1/PB4**	80:20	−0.12	−7.90%
	70:30	−0.1	−6.60%
	50:50	−0.05	−3.30%
	30:70	−0.09	−5.90%
	20:80	−0.16	−10.50%
